# Single-cell spatial immune landscapes of primary and metastatic brain tumours

**DOI:** 10.1038/s41586-022-05680-3

**Published:** 2023-02-01

**Authors:** Elham Karimi, Miranda W. Yu, Sarah M. Maritan, Lucas J. M. Perus, Morteza Rezanejad, Mark Sorin, Matthew Dankner, Parvaneh Fallah, Samuel Doré, Dongmei Zuo, Benoit Fiset, Daan J. Kloosterman, LeeAnn Ramsay, Yuhong Wei, Stephanie Lam, Roa Alsajjan, Ian R. Watson, Gloria Roldan Urgoiti, Morag Park, Dieta Brandsma, Donna L. Senger, Jennifer A. Chan, Leila Akkari, Kevin Petrecca, Marie-Christine Guiot, Peter M. Siegel, Daniela F. Quail, Logan A. Walsh

**Affiliations:** 1 Rosalind and Morris Goodman Cancer Institute, McGill University, Montreal, Quebec Canada; 2 Department of Physiology, Faculty of Medicine, McGill University, Montreal, Quebec Canada; 3 Department of Medicine, Division of Experimental Medicine, McGill University, Montreal, Quebec Canada; 4 Departments of Psychology and Computer Science, University of Toronto, Toronto, Ontario Canada; 5 Department of Human Genetics, Faculty of Medicine, McGill University, Montreal, Quebec Canada; 6 Gerald Bronfman Department of Oncology, McGill University, Montreal, Quebec Canada; 7 Division of Tumor Biology and Immunology, Oncode Institute, Netherlands Cancer Institute, Amsterdam, Netherlands; 8 Department of Diagnostic Radiology, Faculty of Medicine, McGill University, Montreal, Quebec Canada; 9 Department of Medicine, Division of Neurology, King Saud University College of Medicine, Riyadh, Saudi Arabia; 10 Department of Neurology and Neurosurgery, Faculty of Medicine, McGill University, Montreal, Quebec Canada; 11 Department of Biochemistry, Faculty of Medicine, McGill University, Montreal, Quebec Canada; 12 Department of Oncology, University of Calgary, Calgary, Alberta Canada; 13 Arnie Charbonneau Cancer Institute, University of Calgary, Calgary, Alberta Canada; 14 Department of Neuro-Oncology, Netherlands Cancer Institute, Antoni van Leeuwenhoek Hospital, Amsterdam, Netherlands; 15 Lady Davis Institute for Medical Research, Jewish General Hospital, Montreal, Quebec Canada; 16 Department of Pathology and Laboratory Medicine, University of Calgary, Calgary, Alberta Canada; 17 Montreal Neurological Institute-Hospital, McGill University Health Centre, Montreal, Quebec Canada; 18 Department of Pathology, Faculty of Medicine, McGill University, Montreal, Quebec Canada; 19 Department of Anatomy and Cell Biology, Faculty of Medicine, McGill University, Montreal, Quebec Canada

**Keywords:** Cancer microenvironment, CNS cancer, Data acquisition, Tumour immunology

## Abstract

Single-cell technologies have enabled the characterization of the tumour microenvironment at unprecedented depth and have revealed vast cellular diversity among tumour cells and their niche. Anti-tumour immunity relies on cell–cell relationships within the tumour microenvironment^1,2^, yet many single-cell studies lack spatial context and rely on dissociated tissues^3^. Here we applied imaging mass cytometry to characterize the immunological landscape of 139 high-grade glioma and 46 brain metastasis tumours from patients. Single-cell analysis of more than 1.1 million cells across 389 high-dimensional histopathology images enabled the spatial resolution of immune lineages and activation states, revealing differences in immune landscapes between primary tumours and brain metastases from diverse solid cancers. These analyses revealed cellular neighbourhoods associated with survival in patients with glioblastoma, which we leveraged to identify a unique population of myeloperoxidase (MPO)-positive macrophages associated with long-term survival. Our findings provide insight into the biology of primary and metastatic brain tumours, reinforcing the value of integrating spatial resolution to single-cell datasets to dissect the microenvironmental contexture of cancer.

## Abstract (web summary)

Imaging mass cytometry of human brain tumours provides spatial information that, combined with existing transcriptomic data, reveals the existence of a cellular neighbourhood containing a rare macrophage population associated with prolonged survival.

## Main

Brain tumours comprise a diverse repertoire of malignancies that arise either from within the brain or from cancer cells that have spread from other primary sites. The most common types of cancer representing these two classes include glioblastoma (around 50% of all primary brain malignancies in adults^4^) and brain metastasis (BrM) (about 90% of all brain malignancies), with BrM most frequently arising from melanoma, lung or breast tumours^5^. Besides surgery, cytotoxic therapies that target tumour cells—such as stereotactic radiotherapy—are often the first line of treatment, but they yield minimal benefit, with survival beyond 2 years being rare^6,7^. The tumour microenvironment (TME) is a major regulator of cancer progression, whose therapeutic value has grown with the advent of immune checkpoint blockade^1,2^. Compared with other tissues, the brain TME has a distinct composition, dominated by functionally diverse astrocytes and pro-tumorigenic macrophages that are ontogenically distinct, with the exclusion of infiltrating lymphocytes^8^. Many promising therapeutic targets within the TME of other cancers have been revealed by single-cell profiling technologies; for example, multiplex imaging has enabled the discovery of several new biomarkers that are predictive of outcomes and therapeutic efficacy in breast^9–12^, colorectal^13^ and pancreatic cancer^14^. However, comprehensive profiling of the brain TME has seen fewer (albeit important^15–19^) advances compared with other malignancies, and so far none have included the spatial characterization of individual cells within their niche using highly multiplexed histology. Here we use imaging mass cytometry (IMC) on patient samples to characterize the brain TME of glioblastoma and BrM, and explore how spatially resolved features relate to clinical outcomes.

## Mapping the brain TME with IMC

**Fig. 1:**
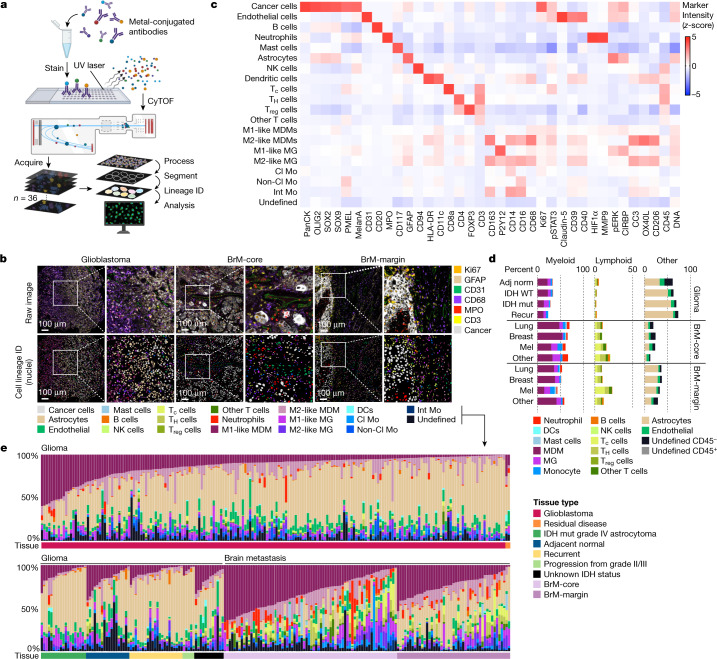
IMC reveals cell dynamics within the brain TME. **a**, Schematic of the IMC pipeline applied to glioma and BrM tissue microarrays. Samples were subject to multiplex staining and data were acquired using cytometry by time-of-flight (CyTOF). Cell segmentation and lineage assignment was performed prior to spatial analysis. Created with BioRender.com. **b**, IMC images from glioblastoma, BrM-core and BrM-margin samples (top) and corresponding lineage assignment (bottom), with magnified regions to the right of each image. The colour codes for IMC markers (top right) and lineage assignment (bottom) are provided (representative of *n* = 389 images). Scale bars, 100 μm. **c**, Heat map showing relative average expression of all markers across cell populations identified using IMC (*n* = 389 images). A subset of markers was specific to the glioma IMC antibody panel (SOX2, SOX9, OLIG2, CD40, CD206; *n* = 270 images) and a second subset to the BrM IMC antibody panel (pan-cytokeratin, PMEL, MelanA, pERK, CIRBP; *n* = 119 images). **d**, Stacked bar graph of the indicated cell types as a percentage of all cells within the TME according to clinical subgroups. Glioma: adjacent normal (adj norm), *n* = 18; primary isocitrate dehydrogenase (IDH) wild type (WT), *n* = 192; primary IDH mutant (mut), *n* = 19; recurrent (recur), *n* = 22. BrM-core: lung, *n* = 29; breast, *n* = 17; melanoma (mel), *n* = 13; other, *n* = 13. BrM-margin: lung, *n* = 22; breast, *n* = 12; melanoma, *n* = 6; other, *n* = 7. Data are mean values; *n* refers to number of images. **e**, The distribution of cell populations as a percentage of all cells in the TME, sorted by tissue type. Cell frequencies for each image (*n* = 389 images) are displayed as vertical bars (colours correspond to cell lineages in **b**) and the associated tissue type is indicated in the horizontal panels below (colours indicated in the legend, right). Cl Mo, classical monocyte; DC, dendritic cells; Int Mo, intermediate monocyte; MG, microglia; Non-Cl Mo, non-classical monocyte; NK cells, natural killer cells; panCK, pan-cytokeratin; T_c_, cytotoxic T cell; T_H_, T helper; T_reg_, T regulatory cell; other T cells, CD8^−^CD4^−^ double-negative T cells.

**Extended Data Fig. 1:**
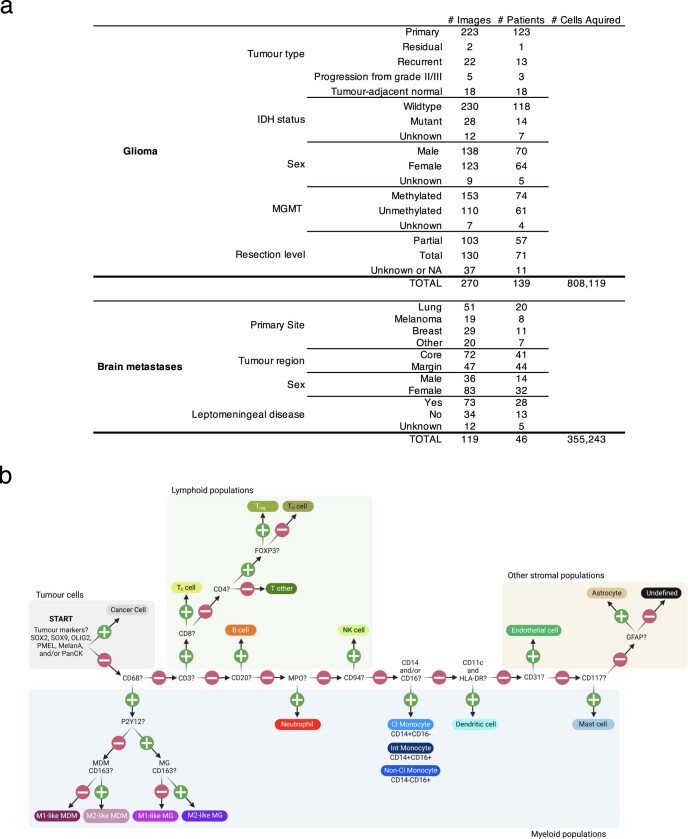
Study cohort and cell lineage assignment. **a**, Summary of patient samples. Patients underwent surgical resection and tumours were classified by a neuropathologist (M.C.G.). Gliomas were classified as: primary (first surgical resection), residual (second surgery to remove any remaining tumour), recurrent (surgical resection after tumour recurrence), or progression from grade II/III (resection of grade IV tumour that progressed from low grade glioma). Glioma samples were extracted from the tumour bulk or the tumour-adjacent normal. Brain metastasis samples were extracted from the tumour bulk (core) or the tumour/brain interface (margin). All clinical information was obtained from surgical and pathological reports. Number of cells acquired corresponds to the total number of cells segmented across all images. **b**, Schematic for cell lineage assignment strategy. Created with BioRender.com.

**Extended Data Fig. 2:**
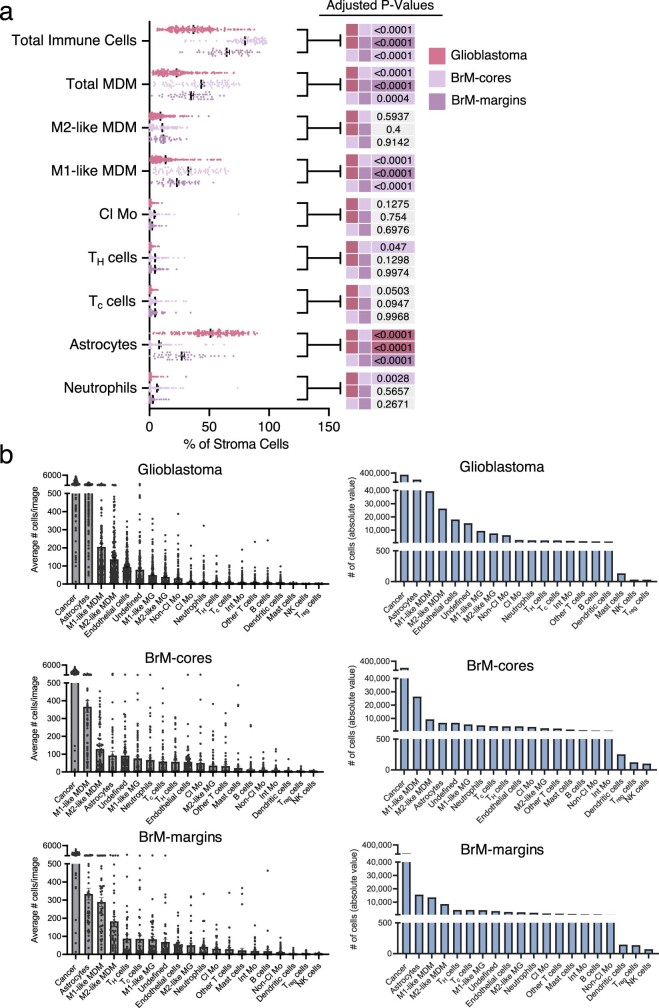
Cellular dynamics in primary & metastatic brain tumours. **a**, Cell frequencies as a percentage of all TME cells in glioblastoma (*n* = 192 images), BrM-cores (*n* = 72 images) and BrM-margins (*n* = 47 images). Data are mean ± s.e.m.; two-way ANOVA; significant adjusted *P*-values are highlighted. **b**, Left: number of cells/image for each cell type in glioblastoma (*n* = 192 images), BrM-cores (*n* = 72 images) and BrM-margins (*n* = 47 images); data are mean ± s.e.m. Right: absolute number of cells (total across all images) in glioblastoma (*n* = 192 images), BrM-core (*n* = 72 images) and BrM-margin (*n* = 47 images).

**Extended Data Fig. 3:**
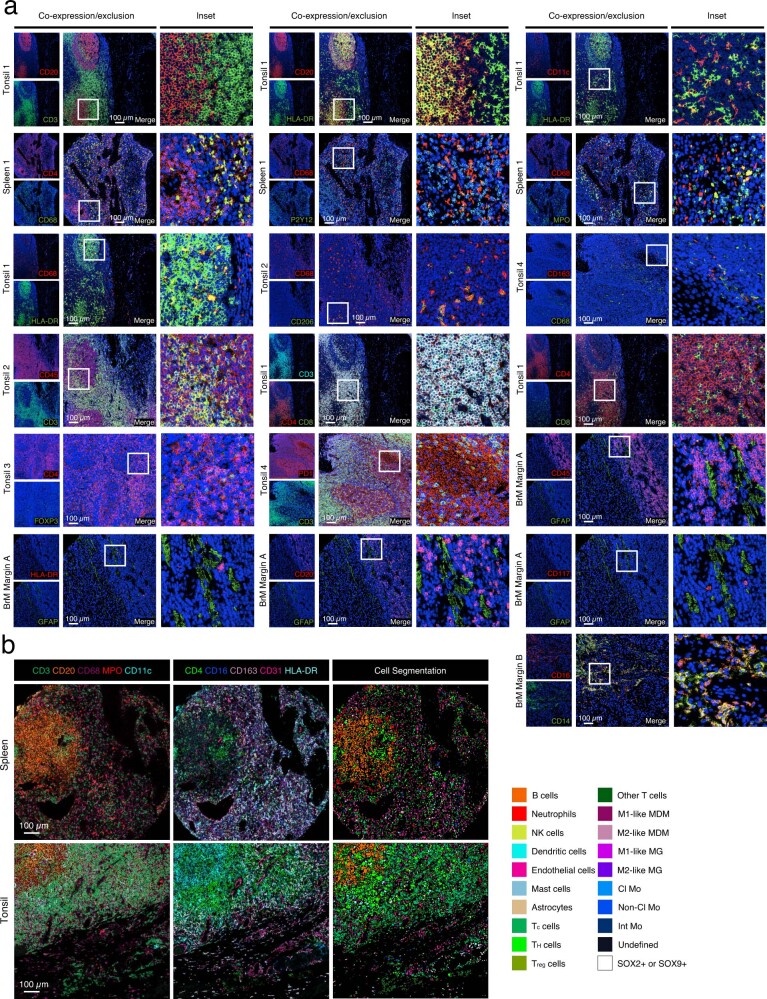
Representative staining of control tissues and brain tumours. **a**, Representative IMC images of expected co-staining patterns in tonsil, spleen or tumour margins. Blue, DNA (191Ir and 193Ir). **b**, Representative IMC images of major lineage markers (left, centre) and corresponding cell segmentation and lineage assignment (right). The colour code for the cell segmentation image is provided (right).

To comprehensively profile the cellular composition and spatial organization of the brain TME, we optimized a highly multiplexed antibody panel and IMC pipeline (Extended Data Figs. 1–3, Supplementary Figs. 1–3 and Supplementary Table 1). Antibodies were validated in normal and malignant tissues on the basis of their expected staining pattern (Extended Data Fig. 3a and Supplementary Figs. 1 and 2). We acquired 389 high-dimensional histopathology images representing 139 high-grade glioma and 46 BrM patient tumours (Fig. 1a,b and Extended Data Fig. 1a). Gliomas comprised resected tissues obtained during surgery (270 images in total, 192 from primary glioblastoma), including a subset from long-term survivors (78 images). BrM images were derived from multiple primary malignancies, including lung (51 images), breast (29 images), melanoma (19 images) and other primary sources (20 images), with patient-matched samples from the centre of the metastatic lesion (BrM-core) and tissue interface (BrM-margin). Images were segmented into 1,163,362 total cells and a supervised lineage assignment approach was used to classify tumour cells, astrocytes, blood vessels and more than 16 immune cell populations using canonical identity markers (Fig. 1c and Extended Data Figs. 1b and 2). As expected, within the stromal compartment across all tissues, major cell populations included GFAP^+^ astrocytes and CD68^+^ macrophages, whereas lymphocytes were relatively infrequent (Fig. 1d,e).

Macrophages stimulated in vitro can be defined along a continuum of activation states; although usually considered M2-like, tumour-associated macrophage activation is much more complex in situ and does not necessarily conform to the M1/M2 paradigm^19,20^. With this limitation in mind, we subdivided macrophages by ontogeny and activation state; expression of the purinergic receptor P2Y12 distinguished tissue-resident microglia from monocyte-derived macrophages^19^ (MDMs), and CD163 expression distinguished putative ‘M2-like’ from ‘M1-like’ cells^21,22^ (Fig. 1c and Supplementary Figs. 3, 4 and 5a). Additional pro-tumorigenic markers, including CD206 and CD39, were also enriched in the CD163^+^ M2-like macrophage fraction (Supplementary Fig. 5b,c). Consistent with previous reports^15,16^, MDMs and microglia were the dominant immune populations across all samples, comprising approximately 30.5% and 9.2% of the TME, respectively (Fig. 1d).

## Single-cell interaction networks

**Fig. 2:**
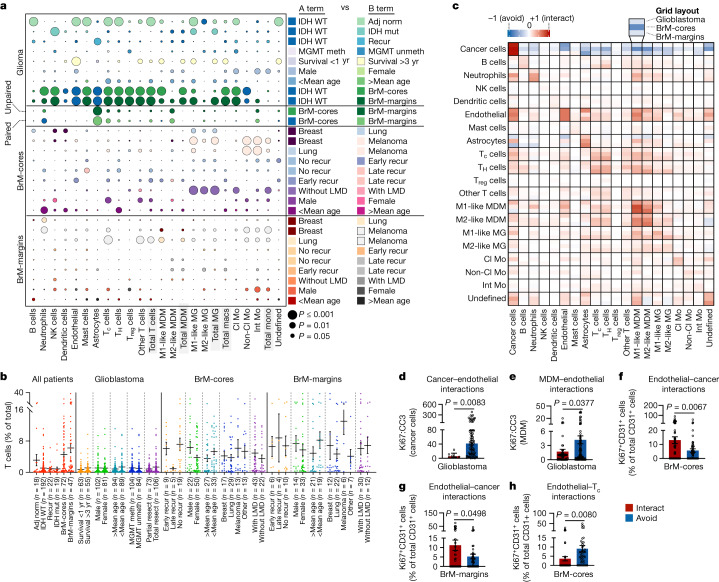
Single-cell interaction networks in high-dimensional histopathology images represent clinical subgroups of patients with brain tumours. **a**, Cell frequency comparisons between clinical subgroups of patients, corresponding to data in **b** and Supplementary Fig. 6. Within each row, the bubble colour indicates the clinical subgroup with the higher cell type representation (A term versus B term, right), and the bubble size indicates the *P-*value. Two-sided Student’s *t*-test, unpaired unless indicated otherwise; paired analyses are from patient-matched samples. LMD, leptomeningeal disease; Meth, methylated; unmeth, unmethylated; recur, recurrence. **b**, T cell frequencies as a percentage of total cells across clinical subgroups. Data are mean ± s.e.m.; all data points overlaid; *n* refers to the number of images. Resect, resection. **c**, Heat map of pairwise interaction–avoidance scores for glioblastoma (top rows, *n* = 192 images), BrM-cores (middle rows, *n* = 59 images) and BrM-margins (bottom rows, *n* = 40 images). Associations should be read row-to-column. **d**, Ki67:CC3 ratio in cancer cells interacting with (red; *n* = 107 cells across 6 images) or avoiding (blue; *n* = 11,163 cells across 67 images) endothelial cells in glioblastoma. Data are median ± interquartile range; two-sided Mann–Whitney test. **e**, Ki67:CC3 ratio in MDMs interacting with (red; *n* = 270 cells across 51 images) or avoiding (blue; *n* = 2,808 cells across 94 images) endothelial cells in glioblastoma. Data are mean ± s.e.m.; two-sided Student’s *t*-test. **f**, Ki67 expression in endothelial cells interacting with (red) or avoiding (blue) cancer cells in BrM-cores. Data are mean ± s.e.m.; *n* = 347 cells across 61 images per group; two-sided Student’s *t*-test. **g**, Ki67 expression in endothelial cells interacting with (red) or avoiding (blue) cancer cells in BrM-margins. Data are mean ± s.e.m.; *n* = 156 cells across 45 images per group; two-sided Student’s *t*-test. **h**, Ki67 expression in endothelial cells interacting with (red) or avoiding (blue) T_c_ cells in BrM-cores. Data are mean ± s.e.m.; *n* = 235 cells across 41 images per group; two-sided Student’s *t*-test. In **d**–**h**, images with zero cells of interest or lacking pairwise interactions of interest were excluded from analysis.

**Extended Data Fig. 4:**
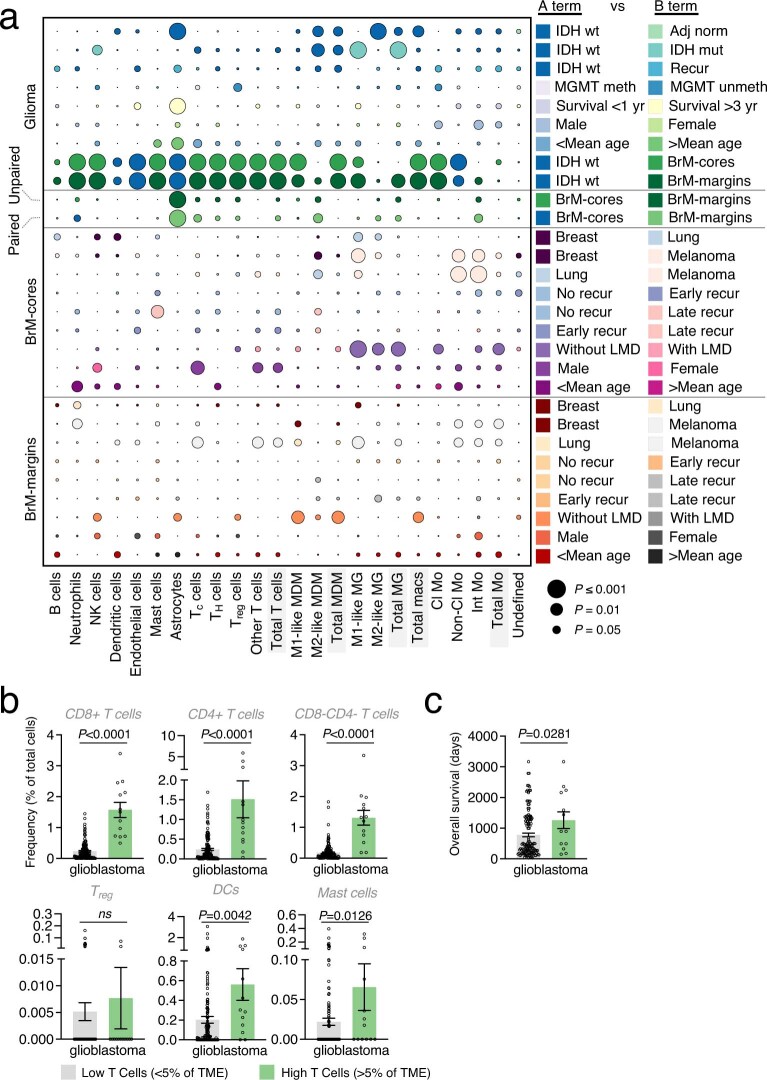
Single-cell frequencies correlate with clinical subgroups in patients with brain tumours. **a**, Cell density comparisons between clinical subgroups of patients, corresponding to Supplementary Fig. 8. Within each row, bubble colour indicates the clinical subgroup (A term versus B term, right) with higher cell type representation; bubble size indicates *P-*value. Two-sided student’s t test, unpaired unless indicated otherwise; paired analyses are from patient-matched samples. **b**, Cell frequencies as a percentage of total cells per image in glioblastoma tumours with low T cells (<5% of TME, *n* = 144 images) versus high T cells (>5% of TME, *n* = 13 images). Data are mean ± s.e.m.; two-sided student’s t test. **c**, Mean overall survival time of glioblastoma patients (for whom this information was available) with low T cells (<5% of TME, *n* = 142 images) versus high T cells (>5% of TME, *n* = 13 images). Data are mean ± s.e.m.; two-sided student’s t test.

We quantified the frequency of each cell type as a percentage of the total number of cells within each image, and compared clinically relevant subgroups of patients (Fig. 2a,b and Supplementary Figs. 6 and 7). Cell density within each image area was similarly assessed (Extended Data Fig. 4a and Supplementary Fig. 8). As expected, there was an increase in the frequency of most cell types in adjacent normal tissue compared with glioblastoma (Fig. 2a), whereas this trend was reversed when examining cell density (Extended Data Fig. 4a), reflecting the sparse cellular landscape of the normal brain niche. In IDH wild-type (glioblastoma) versus IDH mutant (grade IV astrocytoma) tumours^23^, NK cells were reduced in frequency (Fig. 2a). Moreover, there was a lower proportion of CD16^+^ (cytotoxic) than CD16^−^ (immature) NK cell subsets in glioblastoma tumours (Supplementary Fig. 7b,c), consistent with previous findings^15^. We also found a higher frequency and density of recruited MDMs (but not microglia) in IDH wild-type versus mutant tumours (Fig. 2a and Extended Data Fig. 4a), suggestive of enhanced peripheral recruitment of macrophages with greater disease severity^24^. Parsing glioblastoma samples by MGMT methylation status (a prognostic indicator) unveiled minimal immunological differences; however, subdivision by survival time revealed a higher endothelial frequency in tumours from long-term survivors (LTSs) (overall survival more than three years) compared with those from short-term survivors (STSs) (overall survival less than one year) (Fig. 2a). This was unexpected, and may be related to efficiencies in chemotherapy delivery. Alternatively, features of the vascular niche may confer a survival benefit in some patients. Compared with STS tumours, LTS tumours also had a higher frequency of CD8^−^CD4^−^ T cells (potentially including γδ T cells, which are associated with increased survival^25^) and M1-like macrophage accumulation, with no difference in M2-like macrophages (Fig. 2a). Across glioblastoma clinical subgroups, very few lymphocytes were observed based on frequency (Fig. 2b) as well as density (Supplementary Fig. 8), supporting observations that glioblastomas are T cell deserts that exhibit poor responses to immune checkpoint blockade^26^. However, a subset of glioblastoma images exhibited unusually high T cell frequencies (more than 5% of cells in the TME). These samples were enriched for CD8^+^, CD4^+^ and CD8^−^CD4^−^ T cell subsets, but not immunosuppressive T_reg_ cells, and exhibited a 62% increase in mean survival time compared with samples with low numbers of T cells (less than 5% of cells in the TME) (Extended Data Fig. 4b,c). Finally, we observed higher frequencies of peripherally derived monocytes in tumours from male patients than in those from female patients, coinciding with higher frequencies of endothelial cells (Fig. 2a), highlighting putative sexual dimorphism in immune responses to cancer.

We next examined immune cell dynamics across BrM samples. We found an increase in the frequency and density of NK cells (notably, those that were CD16^+^^15^), neutrophils, macrophages, classical monocytes and T cells (including T_reg_ cells^15^) compared with glioblastoma, and a decrease in dendritic cells and non-classical monocytes (Fig. 2a, Extended Data Fig. 4a and Supplementary Fig. 7b,c). When examining immunological changes associated with BrM progression in BrM-cores, we found increases in monocytes and microglia in patients without leptomeningeal disease (Fig. 2a) or local recurrence (after more than 724 days) (Supplementary Fig. 9a), suggesting a putative protective role for these cells in this context. In comparing BrMs arising from distinct primary tumour sites, the degree of BrM-associated inflammation (that is, the frequency of immune cell types analysed) was generally lowest in tumours originating from breast tumours, highest in those from melanoma, with an intermediate level in tumours originating from lung. Melanoma BrM were enriched for monocytes and microglia compared with lung and breast BrM, and exhibited a pronounced accumulation of CD8^+^ T cells in the tumour margin as a percentage of total cells (Fig. 2a and Supplementary Fig. 9b). This is consistent with observations that, unlike glioblastoma, BrM displays some vulnerability to immune checkpoint blockade—particularly melanoma BrM^27,28^. Similar to glioblastoma, we observed sexual dimorphism in BrM-cores, with more CD8^+^ T cells in those from male patients compared with those from female patients. We additionally found more neutrophils, dendritic cells and CD4^+^ T cells in BrM-cores from younger patients compared with those from older patients (Fig. 2a).

**Extended Data Fig. 5:**
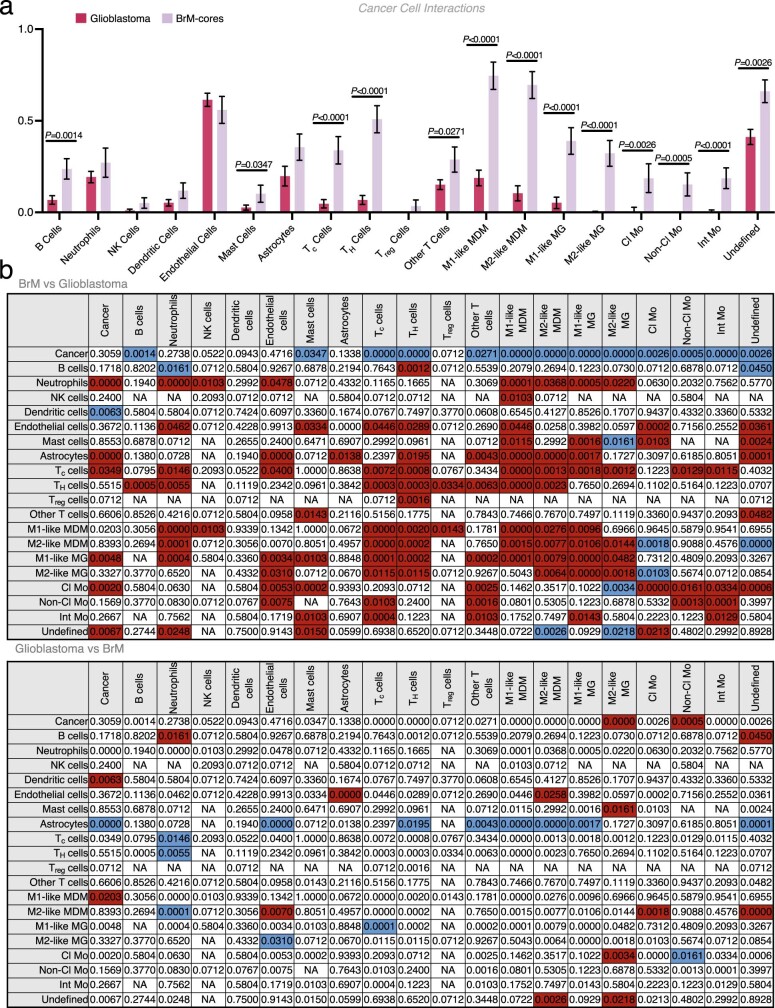
Cellular interactions in primary versus metastatic brain tumours. **a**, Avoidance scores corresponding to Fig. 2c for cancer cell interactions with non-cancer lineages in glioblastoma (*n* = 192 images) and BrM-cores (*n* = 59 images). Data are mean ± s.e.m.; two-sided student’s t test. **b**, Statistical significance of cellular interaction/avoidance scores in glioblastoma (*n* = 192 images) and BrM-cores (*n* = 59 images) corresponding to Fig. 2c. *P*-values calculated by two-sided student’s t test. Colours indicate significantly greater interaction (red) or avoidance (blue) in BrM versus glioblastoma (upper table) or glioblastoma versus BrM (lower table).

To characterize the patterns of communication between individual cells, we interrogated the positional architecture of brain tumours using permutation tests to quantify cell–cell co-localization and identify interaction or avoidance behaviours between cell pairs^11^ (Fig. 2c). This approach revealed that cancer cells in BrM were more likely to avoid most non-cancer lineages within the TME compared with glioblastoma (Fig. 2c and Extended Data Fig. 5a), indicating that the topography of cancer cells in BrM is more compact relative to the dispersed nature of glioblastoma, consistent with the observed pattern of homotypic cellular interactions (Fig. 2c). Global heterotypic cellular interactions were increased in BrM compared with glioblastoma (Fig. 2c and Extended Data Fig. 5b), suggesting that the ways in which glioblastoma and BrM interface with the surrounding brain parenchyma are fundamentally different, despite sharing a common tissue niche.

**Extended Data Fig. 6:**
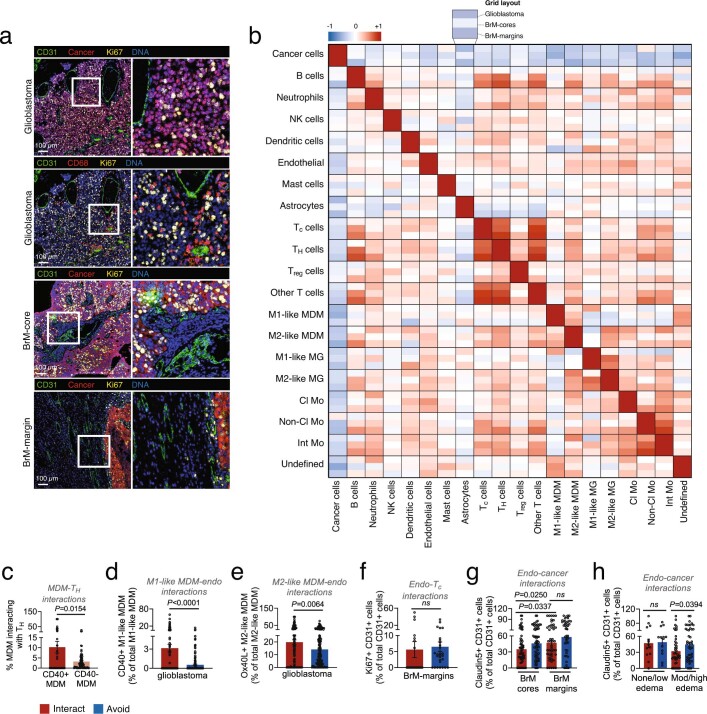
Single-cell spatial interactions in patients with brain tumours. **a**, IMC images of interacting cells in the perivascular niche of glioblastoma and BrM samples, representative of analysis in Fig. 2d–g. First row, perivascular cancer cell proliferation in glioblastoma; second row, perivascular macrophage proliferation in glioblastoma; third row, peritumoural endothelial proliferation in BrM-cores; fourth row, peritumoural endothelial proliferation in BrM-margins. High-magnification insets are provided to the right of each image. **b**, Heatmap of Spearman’s correlation between cell types in glioblastoma (top rows, *n* = 192 images), BrM-cores (middle rows, *n* = 59 images) and BrM-margins (bottom rows, *n* = 40 images). Red, positive correlation; blue, negative correlation. **c**, Frequency of CD40+ MDMs and CD40- MDMs interacting with Th. Data are mean ± s.e.m.; *n* = 1,048 cells across 74 images; two-sided student’s t test. **d**, CD40 in M1-like MDMs interacting with (red) or avoiding (blue) endothelial cells in glioblastoma. Data are mean ± s.e.m.; *n* = 415 cells across 188 images/group; two-sided student’s t test. **e**, Ox40L in M2-like MDMs interacting with (red) or avoiding (blue) endothelial cells in glioblastoma. Data are mean ± s.e.m.; *n* = 4,248 cells across 183 images/group; Two-sided student’s t test. **f**, Ki67 in endothelial cells interacting with (red) or avoiding (blue) Tc cells in BrM-margins. Data are mean ± s.e.m.; *n* = 98 cells across 28 images; two-sided student’s t test. **g**, Claudin-5 in endothelial cells interacting with (red) or avoiding (blue) cancer cells in BrM-cores and -margins. Data are mean ± s.e.m.; BrM-cores, *n* = 1,503 cells across 61 images; BrM-margins, *n* = 1507 cells across 45 images; two-sided student’s t test. **h**, Claudin-5 expression in endothelial cells interacting with (red) or avoiding (blue) cancer cells in BrM-cores subdivided by extent of peritumoural edema. Data are mean ± s.e.m.; none/low edema, *n* = 369 cells across 13 images; moderate/high edema, *n* = 1,046 cells across 42 images; two-sided student’s t test. All images lacking pairwise interactions of interest were excluded from analysis.

The vasculature is a key component of the brain TME in both glioblastoma and BrM; for example, the perivascular niche maintains the glioma-initiating cell pool^29^, and the blood-brain barrier (BBB) regulates BrM dissemination. Given our observation of endothelial enrichment in LTS tumours (Fig. 2a), we examined cellular dynamics within the vascular niche of glioblastoma. As expected, endothelial cells   exhibited a strong likelihood of interacting with astrocytes in glioblastoma, essential for BBB function (Fig. 2c). Endothelial cells also showed strong interactions with cancer cells (Fig. 2c); in particular, perivascular cancer cells exhibited reduced Ki67:CC3 ratios compared with those that avoided blood vessels (Fig. 2d and Extended Data Fig. 6a), suggesting that direct contact with the vasculature may impede cancer cell expansion^30^—a finding that may provide insight into LTS tumour biology. Consistent with previous reports, we observed that MDMs, but not monocytes or microglia, also displayed a strong tendency to interact with endothelial cells in glioblastoma^15,16^ (Fig. 2c), despite a weak correlation between the frequencies of MDMs and endothelial cells (Extended Data Fig. 6b). This highlights that these interactions are spatially coordinated rather than simply resulting from associations in abundance. Mirroring tumour cells, MDMs displayed lower Ki67:CC3 ratios when engaged with the endothelium compared with those that avoided direct endothelial interactions (Fig. 2e and Extended Data Fig. 6a), recapitulating the relationship between proliferating brain macrophages and glioma progression^31^. Finally, despite their low prevalence, there was a modest tendency for T cells to interact with endothelial cells or MDMs (Fig. 2c), prompting us to further dissect these relationships. Specifically, we found more CD40^+^ MDMs interacting with T_H_ cells than CD40^−^ MDMs (Extended Data Fig. 6c), CD40 being a co-stimulatory protein implicated in T cell recruitment in glioma^32^. When examining vessel proximity, perivascular M1-like MDMs exhibited higher CD40 expression than those further away from blood vessels (Extended Data Fig. 6d) and similarly, perivascular M2-like MDMs expressed high levels of OX40L (another co-stimulatory molecule) (Extended Data Fig. 6e). Together, these data allude to the existence of vascular microniches—where macrophages may provide beneficial signalling cues to T cells and cancer cell expansion is kept in check—and support a role for blood vessels in shaping the brain TME contexture.

We next explored vascular interactions in BrM. Similar to glioblastoma, endothelial cells had a high tendency to interact with cancer cells in BrM (Fig. 2c), which is essential for metastatic colonization following extravasation^33,34^. Within both BrM-cores and BrM-margins, endothelial cells that were associated with cancer cells displayed increased Ki67 expression, reminiscent of microvascular proliferation (Fig. 2f,g and Extended Data Fig. 6a). This was of interest given the relationship between microvascular proliferation and high-grade glioma^35^, potentially suggesting localized cellular niches of more aggressive tumour features within metastases. Endothelial cell proliferation appeared to be suppressed via interactions with CD8^+^ T cells—an effect that was specific to BrM-cores (Fig. 2h and Extended Data Fig. 6f). Notably, the expression of claudin-5—a tight junction protein in the BBB implicated in vascular permeability in BrM^36,37^—was spatially regulated. Within BrM-cores (but not BrM-margins), cancer-adjacent endothelial cells exhibited lower claudin-5 expression compared with cancer-avoiding endothelial cells (Extended Data Fig. 6g). Moreover, the frequency of claudin-5^+^ cancer-adjacent endothelial cells was lower in BrM-cores compared with BrM-margins (Extended Data Fig. 6g), supporting a model of vascular co-option during BrM colonization that is initiated in regions of weakened endothelial junctions^34,38,39^. As downregulation of claudin-5 is associated with peritumoural brain oedema^40^, we subdivided BrM-cores by the degree of oedema as assessed by pre-operative MRI. Endothelial cells that were associated with cancer cells displayed reduced claudin-5 in BrMs with a moderate-high degree of peritumoural oedema (oedema score 2–3); this relationship was absent in BrMs with absent or low peritumoural oedema (oedema score 0–1.5) (Extended Data Fig. 6h). These data highlight a spatially resolved link between BBB integrity and metastasis, and how it relates to vascular proliferation and oedema.

## Spatial cellular neighbourhoods

**Extended Data Fig. 7:**
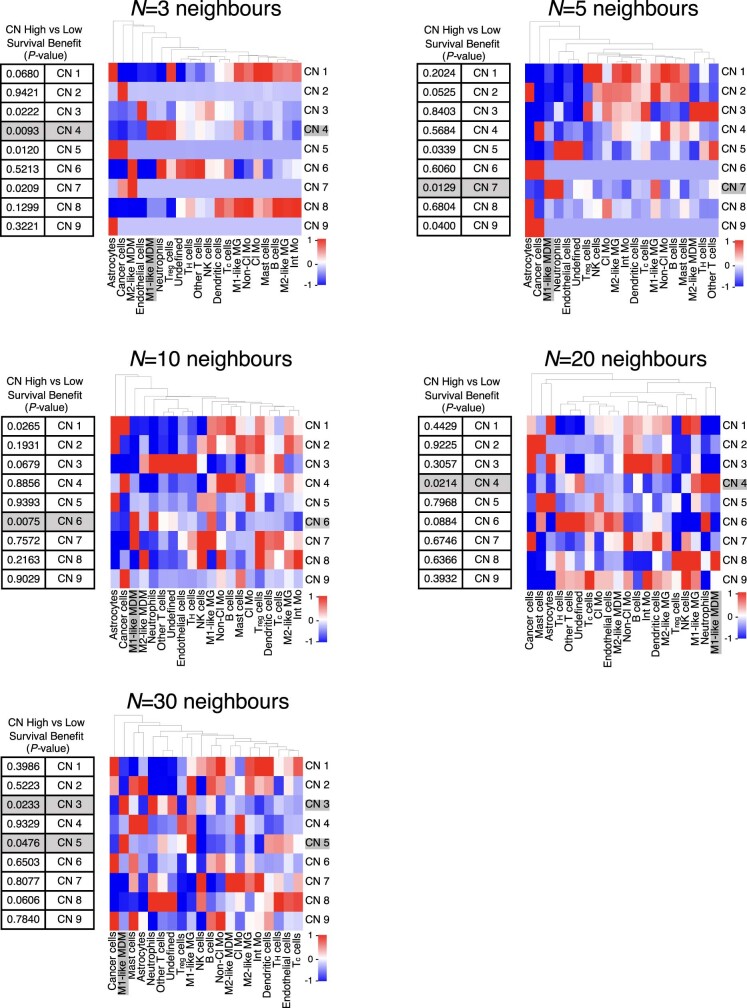
Survival associations of spatial cellular neighbourhoods in glioblastoma. Heatmaps depicting the cellular composition of glioblastoma cellular neighbourhoods (CN), with *N* = 3, 5, 10, 20 or 30 nearest neighbours and CN = 9 neighbourhoods. Tables show *P*-values of survival analyses (Log-rank (Mantel-Cox) test) for samples based on median CN frequency. CNs enriched in M1-like MDM that are significantly associated with survival are highlighted in grey. CN frequencies were averaged when multiple samples corresponded to the same patient.

**Extended Data Fig. 8:**
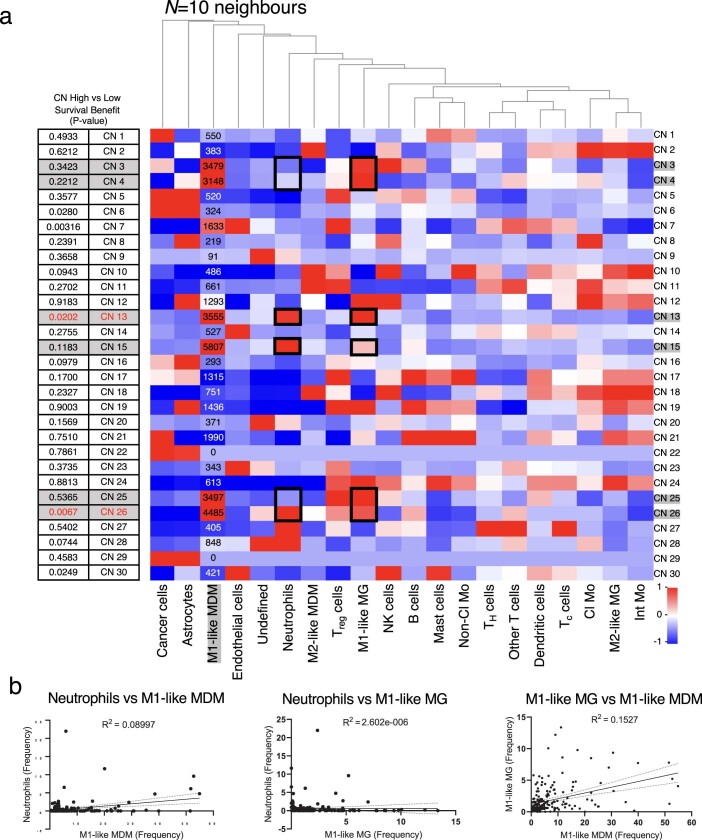
M1-like MDM enriched cellular neighbourhoods are associated with survival in glioblastoma. **a**, Heatmap depicting the cellular composition of glioblastoma cellular neighbourhoods (CN), with *N* = 10 nearest neighbours and CN = 30 neighbourhoods (*n* = 192 images). 6 CNs are enriched with >3000 M1-like MDM (grey highlight; number of M1-like MDM in each CN is indicated). Of these, 2 (red text) are associated with prolonged survival. Table shows *P*-values of survival analyses (Log-rank (Mantel-Cox) test) for samples based on median CN frequency. CN frequencies were averaged when multiple samples corresponded to the same patient. **b**, Cell frequency correlation graphs of neutrophils, M1-like MDM and M1-like MG. Linear regression with 95% confidence interval is shown.

We next explored whether multicellular structures within tumours, rather than pairwise interactions, would provide meaningful insights into the organization and prognostic value of brain TME dynamics. Two variables affect cellular neighbourhood assessment: the number of interacting cells within a neighbourhood (*N*) and the number of total cellular neighbourhoods (CNs). To gain insight into how the size and complexity of neighbourhoods relate to survival, we first used our glioblastoma dataset as a model, and altered the number of nearest spatial neighbours for each individual cell (*N* = 3,5,10,20,30) while maintaining a constant number of neighbourhoods (CN = 9, as in previous work^13^). In most cases, CNs enriched in M1-like MDMs were associated with increased survival, regardless of the number of nearest spatial neighbours (Extended Data Fig. 7). Notably, the frequency of M1-like MDMs was not associated with overall survival (Supplementary Fig. 7a), highlighting the value of spatial relationships rather than abundance alone. To resolve specific cellular interactions that underlie this survival advantage, we forced the number of CNs to 30 (rather than 9) while maintaining *N* = 10 nearest neighbours. Using this approach, we resolved six CNs that were enriched for M1-like MDMs (Extended Data Fig. 8a). Of these, only two maintained their relationship with prolonged survival; these CNs were both primarily composed of M1-like MDMs, neutrophils and M1-like microglia. In the remaining M1-like MDM-enriched CNs, the survival relationship was lost if either neutrophils or M1-like microglia were reduced (Extended Data Fig. 8a). Importantly, we saw no correlation in the prevalence of M1-like MDMs, neutrophils and M1-like microglia (Extended Data Fig. 8b), suggesting that spatial interactions between these cells are purposeful and not a product of their coordinated abundance.

**Fig. 3:**
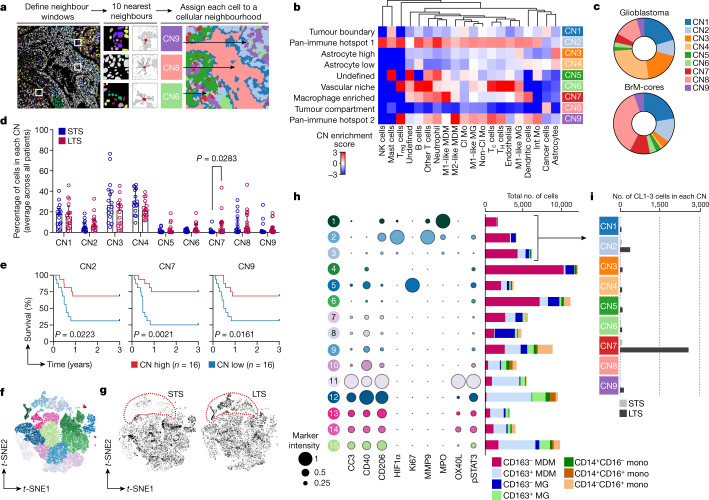
Spatial cellular neighbourhoods relate to survival in glioblastoma. **a**, Schematic of cellular neighbourhood (CN) assignments. CNs are projected as a Voronoi diagram (right). **b**, Heat map of cell types represented across 9 CNs discovered in glioblastoma and BrM-cores (*n* = 251 images; *N* = 10 nearest neighbours, CN = 9 neighbourhoods). **c**, The distribution of CNs across glioblastoma (*n* = 192 images) and BrM-cores (*n* = 59 images). For each image, the percentage of cells from each CN was determined and then averaged for each disease type. **d**–**i**, Analysis of a controlled glioblastoma cohort of LTS (overall survival >3 years) and STS (overall survival <1 year) (see Extended Data Fig. 9f). **d**, The distribution of CNs in the LTS and STS glioblastoma cohort. CN frequencies were averaged where there were multiple samples from the same patient. Data are median ± interquartile range; *n* = 16 patients per group; two-sided Mann–Whitney test. **e**, Kaplan–Meier analysis of the LTS and STS glioblastoma cohort based on the median CN frequency. CN frequencies were averaged where there were multiple samples from the same patient. Log-rank (Mantel–Cox) test; *n* = 16 patients per group. **f**, *t-*SNE unsupervised clustering of macrophages and monocytes from all glioblastoma images (*n* = 93,513 cells across 192 images). **g**, *t-*SNE projection of monocytes and macrophages from patients with glioblastoma, with cells in clusters CL1–3 outlined in red. LTS, *n* = 17,752 cells across 32 images; STS, *n* = 10,456 cells across 28 images. **h**, Relative expression of functional markers (left) and the distribution of cell types (right) across 15 monocyte and macrophage clusters (CL1–15). **i**, The number of cells from CL1–3 found within each cellular neighbourhood in the LTS and STS glioblastoma cohort.

**Extended Data Fig. 9:**
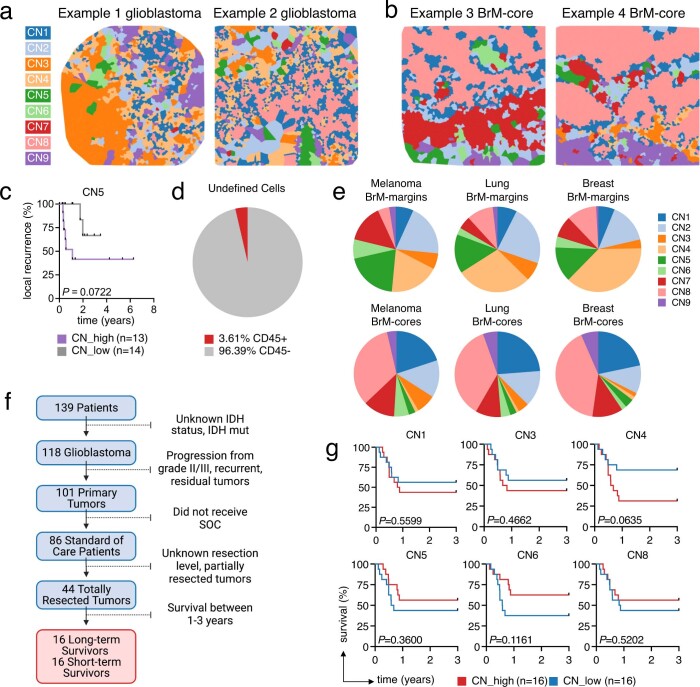
The spatial cellular neighbourhoods of glioblastoma and brain metastases. **a**, Representative Voronoi diagrams of CNs mapped onto glioblastoma IMC images. **b**, Representative Voronoi diagrams of CNs mapped onto BrM-core IMC images. **c**, Kaplan-Meier analysis of BrM patients (lung, melanoma and breast primaries) based on median CN5 frequency in BrM-cores. CN frequencies were averaged when multiple samples corresponded to the same patient. Log-rank (Mantel-Cox) test, *n* = 13 patients/group. **d**, CD45 expression in undefined cells across glioblastoma and BrM samples (*n* = 389 images). **e**, Distribution of CNs across BrM samples. BrM-margins: melanoma *n* = 6 images, lung *n* = 22 images, breast *n* = 12 images; BrM-cores: melanoma *n* = 13 images, lung *n* = 29 images, breast *n* = 17 images. For each image, the percentage of cells from each CN was determined, then averaged for each group. **f**, Schematic of the glioblastoma LTS/STS cohort and inclusion/exclusion criteria, pertaining to Supplementary Table 2. Created with BioRender.com. **g**, Kaplan-Meier analysis of glioblastoma LTS/STS cohort based on median CN frequency. CN frequencies were averaged when multiple samples corresponded to the same patient. Log-rank (Mantel-Cox) test, *n* = 16 patients/group.

We next compared multicellular interactions between glioblastoma and BrM. Using *N* = 10 nearest neighbours (the mid-point of our model and similar to other studies^13^), we identified 9 CNs across glioblastoma and BrM images (Fig. 3a,b and Extended Data Fig. 9a,b). The cellular composition of CNs recapitulated known tissue features, including the tumour boundary (CN1) or tumour compartment (CN8), two pan-immune hotspots with either high levels of all immune populations (CN2) or deficiencies in select subsets (CN9), high (CN3) or low (CN4) astrocytes, vascular niche (CN6), macrophage-enriched (CN7), and a neighbourhood largely represented by cells undefined by our panel (CN5) (Fig. 3b). As expected, glioblastoma was dominated by CN3 and CN4 (astrocyte-enriched) whereas BrM-cores were enriched for CN8 (tumour compartment), reflecting the infiltrative nature of gliomas compared with metastatic tumours (Fig. 3c).

Many patients with BrM exhibit metastatic involvement outside the brain, confounding survival analysis; we therefore correlated CN enrichment with local recurrence. The strongest trend was an association between high CN5 (undefined) and reduced time to local recurrence (Extended Data Fig. 9c). We confirmed that 96% of undefined cells in our dataset were CD45^−^ (non-immune) (Extended Data Fig. 9d). We next investigated the distributions of CNs between BrM-margins and BrM-cores from distinct primary sites. Neighbourhood similarities among BrMs was dictated regionally, rather than by primary tumour type, with BrM-margins being more similar to glioblastoma than BrM-cores (Fig. 3c and Extended Data Fig. 9e). For example, lung BrM-cores were more similar to melanoma or breast BrM-cores than they were to lung BrM-margins (Extended Data Fig. 9e). Despite a high degree of variability in the cell frequencies between BrM from distinct primary tumours (Supplementary Fig. 6), CN distribution was relatively constant, highlighting an ability to identify unifying features in brain tumours that may be therapeutically informative.

We next examined a balanced glioblastoma cohort of STS and LTS by excluding patients with the greatest confounding clinical variable (partial resection) and controlled for other variables that affect survival, such as MGMT methylation status (Extended Data Fig. 9f and Supplementary Table 2). We compared the proportion of cells representing each CN within a given tumour sample, and found that LTS tumours had significantly higher representation of macrophage-enriched CN7 than STS tumours (Fig. 3d). Moreover, using this refined cohort, we confirmed the association between CN7 and improved survival (Fig. 3e and Extended Data Fig. 9g). This aligned with our neighbourhood analysis using variable numbers of interacting cells, where CNs enriched in M1-like MDMs were associated with prolonged survival (Extended Data Fig. 7). Notably, CN2 and CN9 (pan-immune hotspots) were also associated with improved survival (Fig. 3e); analysis of cell dynamics revealed elevated numbers of CD4^+^ T cells in LTS tumours compared with STS tumours (Supplementary Fig. 10), which were enriched in both CN2 and CN9 (Fig. 3b). These data suggest a potential beneficial role for T cell neighbourhoods in glioblastoma, despite their low frequency.

We focused on the prognostic relevance of CN7 in glioblastoma, since it was most consistently associated with survival compared with other CNs. Moreover, targeting macrophages in brain tumours in the clinical setting is receiving increasing interest in light of promising preclinical studies^41–43^. To define macrophage identities across neighbourhoods, we extracted all macrophage and monocyte subsets from glioblastoma samples and performed *t*-stochastic neighbour embedding (*t*-SNE) dimensionality reduction and spectral clustering based on phenotypic markers in our panel (Fig. 3f). Cellular clusters (CL)1–3 were significantly enriched in LTS tumours compared with STS tumours (Fig. 3g and Supplementary Fig. 11), the majority of which were CD163^−^P2Y12^−^, suggestive of an M1-like MDM phenotype (Fig. 3h). However, they co-expressed CD206, indicating these cells do not follow the M1/M2 paradigm, unlike other clusters with high CD206 that were enriched for CD163^+^ cells (for example CL11–15; Fig. 3h). In comparing the relative representation of CL1–3 across each CN (Fig. 3b), we observed an enrichment in CN7 specifically in patients with LTS tumours (Fig. 3i). Together, these data indicate that macrophage spatial relationships may contain critical prognostic information, as we have identified a unique macrophage-enriched neighbourhood associated with long-term survival—a rarity in this disease.

## MPO^+^ macrophages are associated with survival

**Fig. 4:**
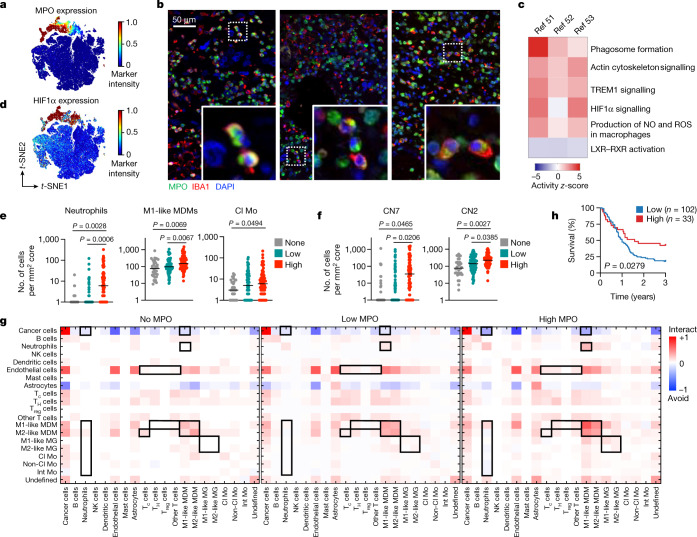
MPO^+^ macrophages are enriched in LTS tumours and are associated with enhanced cytotoxic functions. **a**, Heat map of MPO expression projected onto a *t*-SNE map (Fig. 3f) of monocytes and macrophages from patients with glioblastoma (*n* = 93,513 cells across 192 images). **b**, Representative immunohistofluorescence (IHF) images showing MPO and IBA1 (a macrophage marker) co-localization in glioblastoma tumours. Expanded regions show examples of MPO^+^ macrophages (*n* = 5 images). **c**, Ingenuity pathway analysis of enriched pathways in MPO^+^ versus MPO^−^ macrophages from three publicly available datasets^51–53^. **d**, HIF1α expression projected onto a *t*-SNE map of monocytes and macrophages from patients with glioblastoma (*n* = 93,513 cells across 192 images). **e**, The number of cells per 1 mm^2^ core in glioblastoma samples with zero (*n* = 32 images), low (*n* = 79 images) or high (*n* = 81 images) MPO^+^ M1-like MDMs. The graph shows mean values (black horizontal line) and all data points; one-way ANOVA; data are presented in log scale, so images with 0 cells were assigned a value of 1. **f**, The raw number of cells in each cellular neighbourhood per 1 mm^2^ core from patients with glioblastoma with zero (*n* = 32 images*)*, low (*n* = 79 images) or high (*n* = 81 images) numbers of MPO^+^ M1-like MDMs. The graph shows mean values (black horizontal line) and all data points; one-way ANOVA; images with 0 cells were assigned a value of 1. **g**, Pairwise interactions across two-sided permutation tests on individual images (1,000 permutations each) for patients with zero, low or high numbers of MPO^+^ M1-like MDMs. Red, interactions (interact); blue, avoidances (avoid). **h**, Kaplan–Meier analysis based on MPO^+^IBA1^+^ cell frequency as determined by IHF staining in 135 tumours from patients with glioblastoma (*z*-score). Cell frequencies were averaged when multiple samples corresponded to the same individual. Log-rank (Mantel–Cox) test.

**Extended Data Fig. 10:**
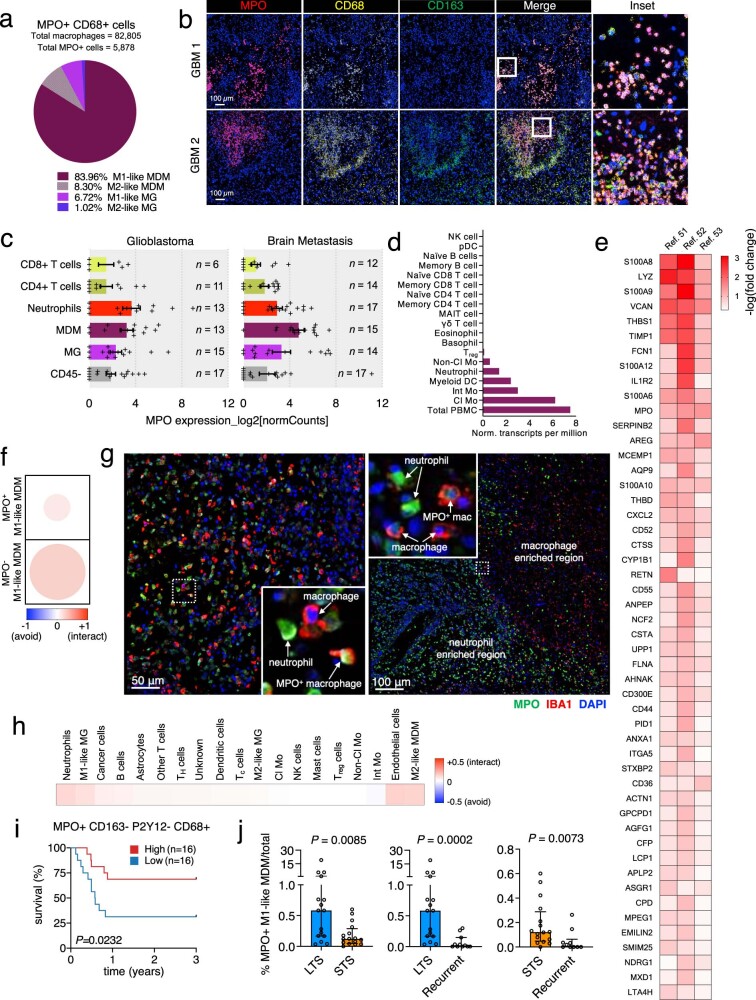
MPO+ macrophages are associated with enhanced cytotoxic functions. **a**, Pie chart depicting the distribution of macrophage subsets within the total pool of MPO+ CD68+ macrophages in glioblastoma (*n* = 192 images). Percentages reflect the proportion of total MPO+ CD68+ cells. **b**, Representative IMC images of MPO+ MDMs in two glioblastoma samples. **c**, Normalized expression of *MPO* in glioblastoma and BrM immune cell subsets via RNA-seq (data from ref. ^16^). Data are mean ± s.e.m.; *n-*values depicted indicate number of patients. **d**, Normalized expression of *MPO* in human blood immune cell subsets via RNA-seq (data from ref. ^61^). **e**, Heatmap displaying the top 50 differentially expressed genes in MPO+ versus MPO- macrophages that were common to three independent publicly available glioblastoma scRNA-seq datasets (data from refs. ^51–53^). **f**, Pairwise interaction/avoidance scores using two-sided permutation tests on individual images (1,000 permutations each) between endothelial cells and MPO+ versus MPO- M1-like MDMs (*n* = 192 images). Colour indicates interaction (red) or avoidance (blue) and circle size reflects the magnitude of the interaction score. **g**, Representative immunohistofluorescence images of MPO (green) and IBA1 (red; macrophage marker) co-localization in glioblastoma tumours. Examples of MPO+ IBA1+ macrophages are shown in insets, along with MPO+ IBA1- neutrophils or MPO- IBA1+ macrophages (*n* = 5 images). **h**, Pairwise interaction/avoidance scores using two-sided permutation tests on individual images (1,000 permutations each) for MPO+ M1-like MDM with other cell types (*n* = 192 images); interaction (red), avoidance (blue). **i**, Kaplan-Meier analysis of glioblastoma LTS/STS cohort based on median MPO+CD163-P2Y12-CD68+ macrophage enrichment. Cell frequencies were averaged when multiple samples corresponded to the same patient. Log-rank (Mantel-Cox) test, *n* = 16 patients/group. **j**, Frequency of MPO+ M1-like MDM as a percentage of total cells in the glioblastoma LTS/STS cohort (*n* = 16 patients/group) and recurrent glioblastoma (n = 10 patients). Frequencies were averaged when multiple samples corresponded to the same patient. Data are median ± interquartile range; two-sided Mann-Whitney test.

Given the relationship between CL1–3 macrophages and LTS tumours, we explored their putative function. CL1–3 macrophages expressed high levels of MPO (Fig. 4a), and more than 80% of MPO^+^ macrophages were CD163^−^P2Y12^−^ (Extended Data Fig. 10a,b), suggesting a pro-inflammatory phenotype dominated by peripherally derived MDMs. MPO is often used as a marker for neutrophils, where it mediates production of reactive oxygen species and oxidative burst. Although it is likely that MPO staining within macrophages partially reflects enhanced phagocytosis of neutrophils, *MPO *transcript is also detectable in brain tumour MDMs (Extended Data Fig. 10c) and peripheral monocytes (Extended Data Fig. 10d) at comparable levels to neutrophils. Indeed, neutrophil-like monocytes and macrophages have been identified in several immunopathologic contexts, including atherosclerosis^44^, neuroinflammation^45,46^ and lung cancer^47^. It is possible they arise either from a shift in monocyte developmental trajectories to favour granulocyte-monocyte progenitor (GMP)-derived lineages^48^ or through MPO induction within the tissue niche. We confirmed the presence of MPO^+^IBA1^+^ macrophages in glioblastoma tumours using immunofluorescence (Fig. 4b; IBA1 was used as an alternative macrophage marker to CD68 for validation purposes). Using single-cell RNA-sequencing datasets from patients with glioblastoma, we identified genes enriched in MPO^+^ macrophages versus MPO^−^ macrophages (Extended Data Fig. 10e). Among the top differentially expressed genes were *S100A8* and *S100A9*, markers of GMP-derived lineages^48,49^. We observed signatures associated with reactive oxygen species biosynthesis and phagosome formation (indicative of cytotoxicity), and HIF1α signalling (suggesting distance from blood vessels) (Fig. 4c). Consistently, MPO^+^ macrophages were less likely to interact with endothelial cells compared with MPO^−^ macrophages (Extended Data Fig. 10f), coinciding with enriched HIF1α in CL1–3 (Fig. 4d). We also saw reduced LXR–RXR signalling (Fig. 4c), suggesting altered fatty acid metabolism within these cells that is consistent with their avoidance of fatty streaks in atherosclerosis^44^. These data suggest that, although rare in the TME, MPO^+^ macrophages may have anti-tumorigenic properties.

We next categorized tissues on the basis of the median density of MPO^+^ M1-like MDM: none, low (1–5 cells per mm^2^) and high (6 or more cells per mm^2^). Higher numbers of MPO^+^ macrophages corresponded with an increase in total neutrophils, M1-like MDMs and classical monocytes (Fig. 4e), supporting the notion that these tumours may be primed for strong innate effector responses. We also compared CN prevalence and found an enrichment in cells associated with CN7 (macrophage-enriched, as expected) and CN2 (pan-immune hotspot) (Fig. 4f), both of which provided a significant survival benefit in patients (Fig. 3e). To gain insight into the tumour architecture, we compared spatial interactions between major immune lineages across MPO categories. Cancer cells displayed a greater tendency to avoid neutrophils and M1-like MDMs as the density of MPO^+^ macrophages increased (Fig. 4g). Indeed, most myeloid cell populations avoided neutrophils as MPO^+^ macrophage density increased, with the exception of M1-like MDMs (Fig. 4g). The relationship between neutrophils and MPO^+^ macrophages was confirmed by immunohistofluorescence, as samples with high MPO^+^IBA1^−^ neutrophils also had high MPO^+^IBA1^+^ macrophages (Extended Data Fig. 10g). Similarly, interaction analysis of MPO^+^ M1-like MDMs revealed interactions with both neutrophils and M1-like microglia (Extended Data Fig. 10h), echoing our CN findings (Extended Data Fig. 8a). Higher densities of MPO^+^ macrophages were also associated with increased interactions between endothelial cells and M1-like MDMs with T cells, concomitant with reduced interactions between M2-like MDMs and CD8^+^ T cells (Fig. 4g), which can be immunoregulatory in cancer. Within the macrophage compartment, associations between MDMs increased, whereas they were relatively less pronounced for microglia (Fig. 4g)—suggesting that the dynamics of tissue-resident versus monocyte-derived macrophages may have distinct effects on tumour biology, as has been suggested in preclinical models^41^. These data illuminate gradual shifts in TME composition with changes in the density of MPO^+^ macrophages.

Finally, to further probe clinical relevance, we confirmed that increased levels of MPO^+^CD163^−^P2Y12^−^CD68^+^ macrophages were associated with prolonged survival using our balanced glioblastoma cohort (Extended Data Fig. 10i). Consistently, recurrent glioblastomas contained fewer MPO^+^CD163^−^P2Y12^−^CD68^+^ macrophages compared with either STS or LTS tumours (Extended Data Fig. 10j). To test the practicality of our findings using a lower-plex technology, we performed immunohistofluorescence co-staining for MPO and IBA1 to evaluate survival outcomes in a cohort of 135 patients with glioblastoma. As the vast majority (83.96%) of MPO^+^ macrophages are CD163^−^ and P2Y12^−^ (Extended Data Fig. 10a), the combination of MPO with IBA1 was sufficient to confirm significantly prolonged survival in patients with high frequencies of double-positive cells (Fig. 4h). Building on previous work showing that macrophage accumulation in brain tumours is associated with advanced disease^24^, our findings highlight an MPO^+^ subpopulation associated with an unexpected survival benefit. This suggests that therapies that broadly target macrophages in glioblastoma may deplete a beneficial macrophage subset, adding insight to clinical trials with CSF-1R inhibitors that have been largely unsuccessful^50^, and deepening our understanding of macrophage complexity in this disease.

## Discussion

We have provided a high-dimensional spatial map of the brain TME using IMC. We performed a comprehensive analysis of cellular dynamics, interactions and neighbourhoods in glioblastoma and BrM, and correlated spatial features of glioblastoma with patient survival. We defined multicellular structures that are common across disease states and regions, which are superior for predicting patient survival compared with cell frequencies alone. We identified a unique subset of neutrophil-like macrophages that stain positively for MPO, which appear to be beneficial for the survival of patients with glioblastoma. We speculate that host immunity favouring GMP-derived neutrophil-like states may be advantageous for patient outcomes^48^. Alternatively, programming macrophages to adopt neutrophil-like characteristics or enhanced phagocytosis may occur within specific tumour niches. Our dataset adds to the growing evidence that the spatial organization of tumours at the cellular level is critical for understanding immunological mechanisms governing cancer. Given the limited therapeutic options for patients with brain tumours and dismal prognosis, there is untapped translational value in understanding how the spatial architecture of the brain TME relates to tumour biology, and whether specific immune cell subsets can be harnessed to improve outcomes.

## Methods

### Clinical samples for IMC

A cohort of 185 patients underwent surgical resection for primary brain tumours or BrM between 2006–2019. A breakdown of the patients whose tumour samples were used in this study can be found in Extended Data Fig. 1a. The clinical data for all patients was obtained from surgical and pathological reports. All tumour samples obtained from patients with glioblastoma were previously untreated and classified by a certified neuropathologist (M.C.G.) following primary surgical debulking. A subset of tumours was removed during a second follow-up surgery (residual tumour) or following tumour recurrence (recurrent tumour). In accordance with World Health Organization 2021 guidelines^23^, tumours formerly classified as IDH mutant glioblastoma are now considered grade IV IDH mutant astrocytoma; therefore, in this study, only grade IV IDH wild-type tumours were designated as glioblastoma. We further distinguished glioblastomas that resulted from progression from grade II/III tumours (Extended Data Fig. 1a). LTSs were defined as patients with an overall survival greater than three years (much longer than expected survival time), and STSs were defined as patients with an overall survival less than one year (shorter than expected survival time). Brain metastases samples^54^ were surgically removed from patients bearing lung (*n* = 51 images), breast (*n* = 29 images), and melanoma (*n* = 19 images) primary tumours as well as a small number of bladder, colorectal, gastric, gastrointestinal and ovarian tumours, collectively called ‘other’ (*n* = 20 images) in this study. Pre-operative MRI images were used to determine the extent of peritumoural oedema (scored 0–3) by a neuroradiologist (S.L.). Leptomeningeal disease^55^ was determined by contrast-enhancing lesions in the subarachnoid or ventricles as determined on MRI by a neuroradiologist (S.L.). All patients underwent standard of care (SOC) following surgery, unless otherwise specified. Cores (1–1.5 mm in diameter) were removed from formalin-fixed, paraffin embedded (FFPE) tissue blocks and assembled into tissue microarrays (TMAs). Within the glioma cohort, we included tumour-adjacent ‘normal’ tissues as well as primary brain tumour samples extracted from the tumour bulk, which were confirmed using corresponding haematoxylin and eosin (H&E) staining by a neuropathologist (M.C.G.). BrM samples were extracted from the tumour bulk and/or tumour–brain interface (termed BrM-cores and BrM- margins, respectively). A total of 242 tissue regions were sampled across the 185 patients including 139 high-grade glioma, 18 glioma-adjacent normal, 41 BrM-cores and 44 BrM-margins. Of these 242 regions, 142 were sampled in duplicate and 2 were sampled in triplicate for a total of 389 cores. Additionally, 39 patients with BrM had matched core-margin pairs. All surgical specimens and clinical information were obtained following written informed patient consent. Clinical information was de-identified and used in accordance with the institutional review boards of McGill University and Montreal Neurological Institute-Hospital (REB: NEU-10-066, 2018-4150).

### Antibody optimization

Antibodies were optimized on control tissues including spleen, tonsil, lymph node, liver, kidney, normal lung, normal brain, lung cancer, glioblastoma and/or BrM. In Extended Data Fig. 3a and Supplementary Figs. 1 and 2, we show representative optimization images of both immunohistofluorescence (IHF) and IMC staining for all markers in our panel, with some exceptions: IHF was not performed for antibodies that were commercially available with conjugated metal isotopes (except CD20 and CD45; unconjugated forms were used for IHF), and IHF was not performed for Ki67 as the B56 clone is routinely used. A list of all antibodies can be found in Supplementary Table 1. For IHF staining, FFPE sections underwent deparaffinization and heat-mediated antigen retrieval using the Ventana Discovery Ultra auto-stainer platform (Roche Diagnostics) according to manufacturer instructions. FFPE slides were incubated at 70 °C in pre-formulated EZ Prep solution (Roche Diagnostics), followed by incubation at 95 °C in pre-formulated Cell Conditioning 1 solution (Roche Diagnostics) for a total run time of ~2.5 h. Slides were rinsed in 1× PBS and incubated for 1 h at room temperature in Dako Serum-free Protein Block solution (Agilent). An antibody cocktail was prepared in Dako Antibody Diluent and slides were incubated with primary antibodies overnight at 4 °C. Slides were rinsed with 1× PBS and incubated with secondary antibody cocktail prepared in Dako Antibody Diluent for 1 h at room temperature. Slides were counterstained with DAPI for 5 min at room temperature and mounted using Dako Mounting Medium. An AxioScan Z1 scanner was used to capture tissue images.

### Immunostaining and IMC

FFPE TMA slides underwent deparaffinization and heat-mediated antigen retrieval using the Ventana Discovery Ultra auto-stainer platform (Roche Diagnostics) according to the manufacturer’s instructions. FFPE slides were incubated at 70 °C in pre-formulated EZ Prep solution (Roche Diagnostics), followed by incubation at 95 °C in pre-formulated Cell Conditioning 1 solution (Roche Diagnostics) for a total run time of ~2.5 h. Slides were rinsed in 1× PBS and incubated for 45 min at room temperature in Dako Serum-free Protein Block solution (Agilent). An antibody cocktail containing metal-conjugated antibodies was prepared in Dako Antibody Diluent at optimized dilutions. Slides were stained with primary antibodies at 4 °C overnight and subsequently washed with 0.2% Triton X-100 and 1× PBS. A secondary antibody cocktail containing metal-conjugated anti-biotin was prepared in Dako Antibody Diluent at the optimized dilution. Slides were incubated with anti-biotin for 1 h at room temperature and subsequently washed with 0.2% Triton X-100 and 1× PBS. Slides were counterstained with Cell-ID Intercalator-Ir (Fluidigm) diluted at 1:400 in 1× PBS for 30 min at room temperature, rinsed for 5 min with distilled water, and air-dried prior to IMC acquisition. IMC acquisition was performed using the Hyperion Imaging System (Fluidigm).

### Data transformation and normalization

All IMC data presented were not transformed and analyses were based on raw measurements. Single-cell marker expressions are summarized by mean pixel values for each channel. For heat map visualization, expression data were normalized to the 95th percentile and *z*-scored cluster means were plotted.

### Cell segmentation and lineage assignment

All lineage and functional markers underwent a staining quality check prior to cell segmentation. A subset of functional markers passed initial quality control, but did not stain consistently with IMC, and were subsequently removed from analysis (GM-CSF-R, M-CSF-R, PD-1, PD-L1 and CTLA-4; see Supplementary Fig. 1). Cell segmentation was done using a combination of classical and modern machine learning-based computer vision algorithms. This pipeline enables high-throughput segmentation and accurately resolves individual cells across diverse tissues and structures. Importantly, this algorithm fully automates the detection of cells, thus eliminating subjective bias. The DNA channel is pre-processed for nuclei segmentation to obtain foreground regions of interest using mixtures of generalized Gaussian distributions (MoGG). The channels are also tiled for segmentation so we can pass them as inputs for inference to the MaskRCNN model. A detailed description of our segmentation and image analysis pipeline is available^56^. To assign cell phenotypes, we established a supervised approach based on canonical lineage markers, expected population abundance, staining quality, and maturity of cell lineage (Extended Data Fig. 1b). We first used *k*-means clustering^57^ and a mixture of generalized Gaussian models^58^ to create multi-level image stacks based on the staining intensity of each marker. Masks were curated for each lineage marker in the panel based on consideration of 6 levels using the following procedure. (1) Greyscale image channel is convolved with a median filter with a 3 × 3 window size. (2) Each pixel is clustered into 6 groups of intensity levels using the *k*-means algorithm. (3) For each channel we select all groups up to a particular level as foreground (1) and the rest are designated as background (0). (4) We apply morphological blob removal to obtain smoother binary masks, where binary blobs of a particular area are removed from masks to avoid noisy regions. (5) To further refine the accuracy of select markers, additional channel-specific morphological operations are applied by computing an additional binary mask obtained using the adaptive binarization method with a sensitivity of 0.4. This mask is then amalgamated with the mask obtained in step 4. (6) To enhance the image intensity of select channels, we apply a simple contrast enhancement filter by saturating the bottom and top intensity levels of pixels in particular channels. This process enables us to capture more accurate masks from channels when phenotyping cells within our cores.

The method of lineage assignment is represented in the following formula: for each cell *c*_*i*_ we consider the curated mask for each lineage marker *M*_*k*_, where *k* = 1,…,*n* and *n* is the number of lineage markers. Let us assume ${p}_{{c}_{i}}^{j}$ be the *j*^th^ pixel that lies in the surrounding of *c*_*i*_ and each pixel has the following presence vector based on the lineage markers:

$$E\left(({p}_{{c}_{i}}^{j})\right)=\{{p}_{{M}_{1}}^{j},{p}_{{M}_{2}}^{j},\ldots ,{p}_{{M}_{n}}^{j}\}$$

where ${p}_{{M}_{i}}=\{0\,\text{or}\,1\}$, which determines whether the pixel ${p}_{{c}_{i}}^{j}$ is positive for a particular marker. Next, to determine whether each pixel within a cell is positive or negative for a given marker, we determine the majority vector by summing over the presence of all vectors as:

$${M}_{{c}_{i}}=\left\{\mathop{\sum }\limits_{j=1}^{{N}_{{c}_{i}}}{p}_{{M}_{1}}^{j},\mathop{\sum }\limits_{j=1}^{{N}_{{c}_{i}}}{p}_{{M}_{2}}^{j},\ldots ,\mathop{\sum }\limits_{j=1}^{{N}_{{c}_{i}}}{p}_{{M}_{n}}^{j}\right\}$$

where ${N}_{{c}_{i}}$ is the number of pixels in the cell *c*_*i*_. The maximum value in vector ${M}_{{c}_{i}}$ determines the cell type assignment. Cell lineages are assigned in rank priority order (Extended Data Fig. 1b).

All code used to perform these analyses is available at https://github.com/walsh-quail-labs/IMC-Brain.

### Cell–cell pairwise interaction analysis

To identify significant pairwise interaction and avoidance behaviours between cell types, we performed permutation tests of single-cell interactions as previously described^11,59^. Cells within a 6 pixel radius (6 µm) were considered interacting. Significant interaction or avoidance behaviours were defined as having a *P*-value of less than 0.01.

### Cellular neighbourhood identification

To identify spatial cellular neighbourhoods, we first computed neighbour windows, which we defined as being the number (*N*) of nearest cells to each cell (as indicated), as previously described^60^. Each window is a frequency vector consisting of the types of *N* closest cells to a given cell. Neighbour windows were clustered. Cellular neighbourhood discovery on glioblastoma and BrM-cores combined (performed in 2021) was performed using Scikit-learn, a software machine learning library for Python. Clustering was performed using MiniBatchKMeans clustering algorithm version 0.24.2 with default batch size = 100 and random_state = 0. BrM-margins were excluded from cellular neighbourhood discovery due to their variable mix of tumour versus stromal content. Cellular neighbourhood analysis on glioblastoma cores alone (performed in 2022) used MiniBatchKMeans clustering algorithm version 1.1.2 with default batch size = 1,024 and random_state = 0. Every cell was then assigned to a particular cellular neighbourhood based on their neighbour window. Cellular neighbourhood prevalence in each core was normalized so the sum of cellular neighbourhood prevalence for that core was 100%. Values were then *z*-scored and cores with *z*-score above or equal to 0 and below 0 were compared for survival outcome.

### IMC survival analysis

Glioblastoma survival analysis was conducted using a clinically controlled cohort of patients that received gross total resection of the tumour prior to treatment, as confirmed by post-surgical MRI, and were treated with SOC (Extended Data Fig. 9f and Supplementary Table 2). Overall survival was calculated from the date of surgery to date of death. For patients with glioblastoma whose date of death was not specified, overall survival was estimated using the date of their last known follow-up. For BrM survival analyses, local recurrence-free survival was assessed in previously untreated lesions with complete macroscopic gross total resections as confirmed by post-surgical MRI. For all Kaplan–Meier analyses, images were averaged when multiple cores were collected from the same patient’s tumour (that is, each patient had only one survival value represented in the analysis).

### *t*-SNE

Using default parameters, *t*-SNE dimensionality reduction plots were generated in MATLAB (version 2019b). Clustering was performed using a customized high-dimensional spectral-based clustering algorithm, due to the curse dimensionality of our cells (order of million number of cells). In our customized algorithm, we first use the DBSCAN to isolate clusters that have a similar density (with fixed parameters of a maximum distance of 3 pixels minimum number of 30 points per cluster). This approach produces some small and some big clusters with densities that are similar to each other. The big cluster group is then re-clustered using a spectral clustering algorithm. To be able to achieve a spectral clustering result on our massive dimensional data, we do a subsampling of the data (with a subsampling rate of 10), which gives us the overall shape of the data. Next, we assign each cluster with its cluster labels obtained from spectral clustering. Finally, we fit a *k*-nearest neighbour classifier (with *k* = 5) to our labelled subsampled data, to identify the cluster labels of all samples. Markers used for *t*-SNE analysis include CD14, CD16, CD68, CD163, P2Y12, CC3, Ki67, CD40, CD206, HIF1α, MMP9, MPO, OX40L and pSTAT3. For visualization, expression data were normalized to the 95th percentile.

### Immunohistofluorescence co-staining

FFPE tissue sections were deparaffinized and underwent heat-mediated antigen retrieval in citrate buffer pH 6.0 or EDTA buffer pH 9.0. Slides were blocked with Power Block for 5 min at room temperature and incubated with the primary antibody for 30 min at room temperature. Slides were rinsed with TBS-T and subsequently incubated with secondary antibody–horseradish peroxidase (HRP) for 30 min at room temperature. Slides were rinsed with TBS-T and stained with Opal fluorophore working solution for 10 min (AKOYA Biosciences; Opal 520: FP1487001KT, lot 202212718; Opal 570: FP1488001KT, lot 20212821). This was followed by heat-mediated antibody stripping to remove primary and secondary antibodies. These steps were repeated for each primary antibody for a total of two rounds of labelling: MPO, Abcam, EPR20257, ab208670, lot GR3390666-13, 1:500; IBA1, Fujifilm Wako Pure Chemicals, polyclonal, 019-19741, lot 41375175, 1:400; and Horse Anti-Rabbit IgG HRP Polymer Kit, Vector Laboratories, MP-7801, lot ZH0611, 1:1.

Antibody specificities and dilutions were optimized individually before multiplexing was performed. Tissue images were captured using the AxioScan Z1 scanner and processed using HALO software (version 3.5).

### Clinical samples for IHF

Tissue microarrays containing glioblastoma primary tumour samples from n = 135 patients were consolidated from McGill University (Quebec, Canada), University of Calgary (Alberta, Canada) and the Netherlands Cancer Institute (NKI, Amsterdam, The Netherlands). All patients were previously untreated and classified as IDH wild-type glioblastoma by a certified neuropathologist following primary surgical debulking, and later treated with SOC. All patient information and tissues were obtained after written informed consent and used in accordance with the following ethics oversight.

#### McGill University cohort

*n* = 70 patients underwent surgical resection between 2006–2019; McGill University Health Centre and the Montreal Neurological Institute and Hospital institutional review boards (NEU-10-066, 2018-4150); a neuropathologist reviewed all cases and provided the TMA (M.C.G.). These samples represent a subset of our original IMC cohort based on tissue availability on the TMA (an independent section was used). Survival <1 year, *n* = 25 patients; survival 1–3 years, *n* = 18 patients; survival >3 years, *n* = 27 patients.

#### University of Calgary cohort

*n* = 58 patients underwent surgical resection between 2002 and 2020; Health Research Ethics Board of Alberta, Cancer Committee (HREBA.CC-16-0762), Clark Smith Tumour Biobank; a neuropathologist reviewed all cases and provided the TMA (J.A.C.). Survival <1 year, *n* = 31 patients; survival 1–3 years, *n* = 23 patients; survival >3 years, *n* = 4 patients.

#### NKI cohort

*n* = 7 patients; Institutional Review Board of the NKI-AvL and NKI-biobank (CFMPB541); TMA provided by D.B. Survival <1 year, *n* = 5 patients; survival 1–3 years, *n* = 1 patient; >3 years, *n* = 1 patient.

### Publicly available RNA-sequencing data

Single-cell RNA-sequencing datasets were downloaded from the following.

1. GSE154795 (ref. ^51^) (GSE154795_GBM.AllCell.Integrated.Scaled.ClusterRes.0.1.rds.gz). Using the Seurat object file GSE154795_GBM.AllCell.Integrated.Scaled.ClusterRes.0.1.rds, a new Seurat object was created (Seurat 4.1.1), with the RNA assay counts from the subset of the 14 new patients with glioblastoma and was normalized with the default parameters of the Seurat function NormalizeData.
2. GSE162631 (ref. ^53^) (GSE162631_raw_counts_matrix.zip.gz). A Seurat object was created using Seurat 4.1.1 from the expression matrix count files with the parameters min.cells = 0 and min.features = 200. The counts were normalized with the default parameters of the Seurat function NormalizeData.
3. 10.17605/OSF.IO/4Q32E^52^. Using the Seurat object file seurat.obj_MNN_ref.RDS, a new Seurat object was created using Seurat 4.1.1 with the RNA assay counts of the source Seurat object and were normalized with the default parameters of the Seurat function NormalizeData.

MDMs from each dataset were characterized as *CD68*^high^ (normalized expression >0) and *P2RY12*^low^ (normalized expression <0.1) and were isolated for further downstream analysis. MDMs were subdivided by *MPO*^high^ (normalized expression >0.05) or *MPO*^low^ (normalized expression <0.05). For each individual patient, an average expression matrix was generated from the *MPO*^high^ and *MPO*^low^ MDMs. The FindMarkers function in Seurat was used to generate a list of differentially expressed genes between the *MPO*^high^ and *MPO*^low^ MDMs. Pathway enrichment was assessed using Ingenuity Pathway Analysis software v.01–13 (Qiagen). Differentially expressed genes (adjusted *P* < 0.05) were selected for each dataset and ‘Core Analysis’ was used with all default parameters.

The following data were also used. Transcriptomic data from human immune cells in blood^61^, accessed via Human Protein Atlas (https://www.proteinatlas.org/ENSG00000005381-MPO/immune+cell); glioblastoma RNA-sequencing data from The Cancer Genome Atlas (TCGA Firehose Legacy), accessed via the cBioPortal for cancer genomics (https://www.cbioportal.org); and bulk RNA-sequencing data from sorted immune cells from brain tumours^16^, accessed via https://joycelab.shinyapps.io/braintime/.

### Statistics and reproducibility

All image analysis steps were performed in MATLAB (version 2019b) and Python (version 3.7.12). Statistical analyses were performed using GraphPad Prism 9 statistical software. *P-*values of <0.05 were considered significant and data were expressed as mean ± s.e.m. unless indicated otherwise in the figure legends. Normal distribution was examined via the Shapiro–Wilk test. Parametric data were analysed by Student’s *t*-test, one-way or two-way ANOVA. Non-parametric data were analysed by Mann–Whitney test; for large sample size comparisons, Student’s *t*-test was used^62^. Survival data were analysed by log-rank (Mantel–Cox) tests, as indicated in the relevant figure legends. Contingency tables were analysed by Fisher’s exact test. Tukey’s test was used for multiple comparisons. For all analyses related to survival, including Kaplan–Meier analysis and the LTS and STS cohort, images were averaged when multiple cores were collected from the same patient’s tumour to prevent biasing results toward individuals with more images. All other analyses unrelated to survival (for example, population dynamics) were performed using individual images to appropriately capture heterogeneity within the TME. Area analysis of IMC images was performed using ImageJ (version 1.53k). All antibody optimization was repeated at least two times by IHF and an additional two times by IMC, using a broad variety of tissues as shown in Extended Data Fig. 3 and Supplementary Fig. 1. Additional representative IMC images (including Fig. 1b, Extended Data Figs. 6a and 10b and Supplementary Fig. 3) were selected from 389 total images and depict the statistical changes and/or staining quality as described; similar results in staining quality were obtained for all samples included in analysis. All other representative immunostaining (Fig. 4b, Extended Data Fig. 10g and Supplementary Fig. 2) was performed on at least five full tissue samples with similar results.

### Reporting summary

Further information on research design is available in the Nature Portfolio Reporting Summary linked to this article.

## Online content

Any methods, additional references, Nature Portfolio reporting summaries, source data, extended data, supplementary information, acknowledgements, peer review information; details of author contributions and competing interests; and statements of data and code availability are available at 10.1038/s41586-022-05680-3.

## Supplementary information

Supplementary Information This file contains representative images and other panel validation data (Supplementary Figs. 1–5), analysis of cell frequencies and densities across clinical groups (Supplementary Figs. 6–11), and antibody and patient cohort information (Supplementary Tables 1 and 2).

Reporting Summary

## Data Availability

The source data supporting findings in this study, including high-dimensional TIFF images and clinical information corresponding to IMC, have been deposited at 10.5281/zenodo.7383719. Raw primary imaging data can be obtained from the authors directly upon reasonable request.

## Code availability

All original code has been deposited at GitHub and can be accessed at: https://github.com/walsh-quail-labs/IMC-Brain.
