# Supplementary material for: Single-cell spatial immune landscapes of primary and metastatic brain tumours
Source: Nature. 2023 Feb 1;614(7948):555–63. doi: 10.1038/s41586-022-05680-3 (PMC9931580; doi:10.1038/s41586-022-05680-3)
Supplement: Supplementary file 1 — This file contains representative images and other panel validation data (Supplementary Figs. 1–5), analysis of cell frequencies and densities across clinical groups (Supplementary Figs. 6–11), and antibody and patient cohort information (Supplementary Tables 1 and 2). [file 41586_2022_5680_MOESM1_ESM.pdf]

nature portfolio

https://doi.org/10.1038/s41586-022-05680-3

Supplementary information

Single-cell spatial immune landscapes of primary and metastatic brain tumours

In the format provided by the authors and unedited

Supplementary Fig. 1

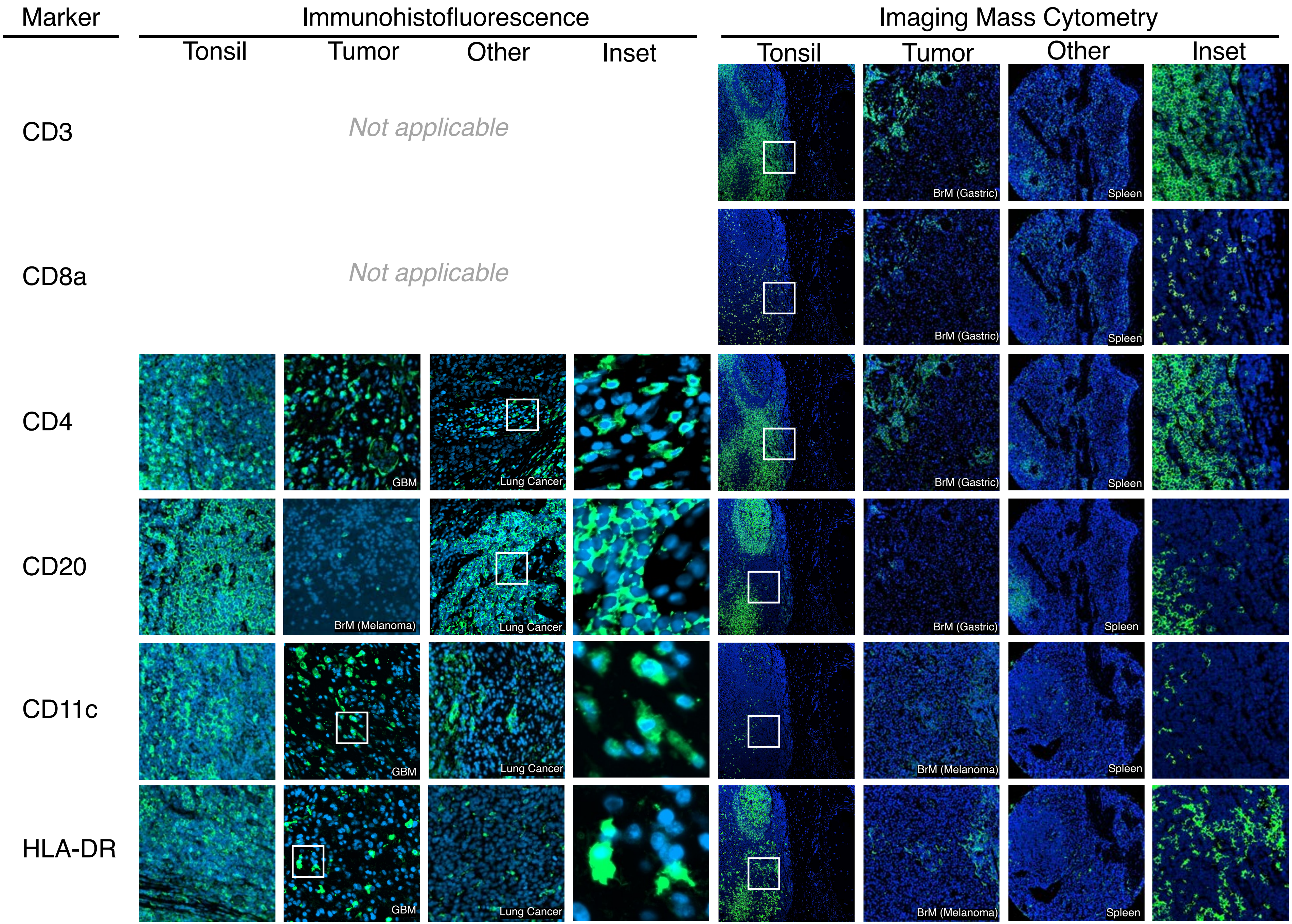

Supplementary Fig. 1 (continued)

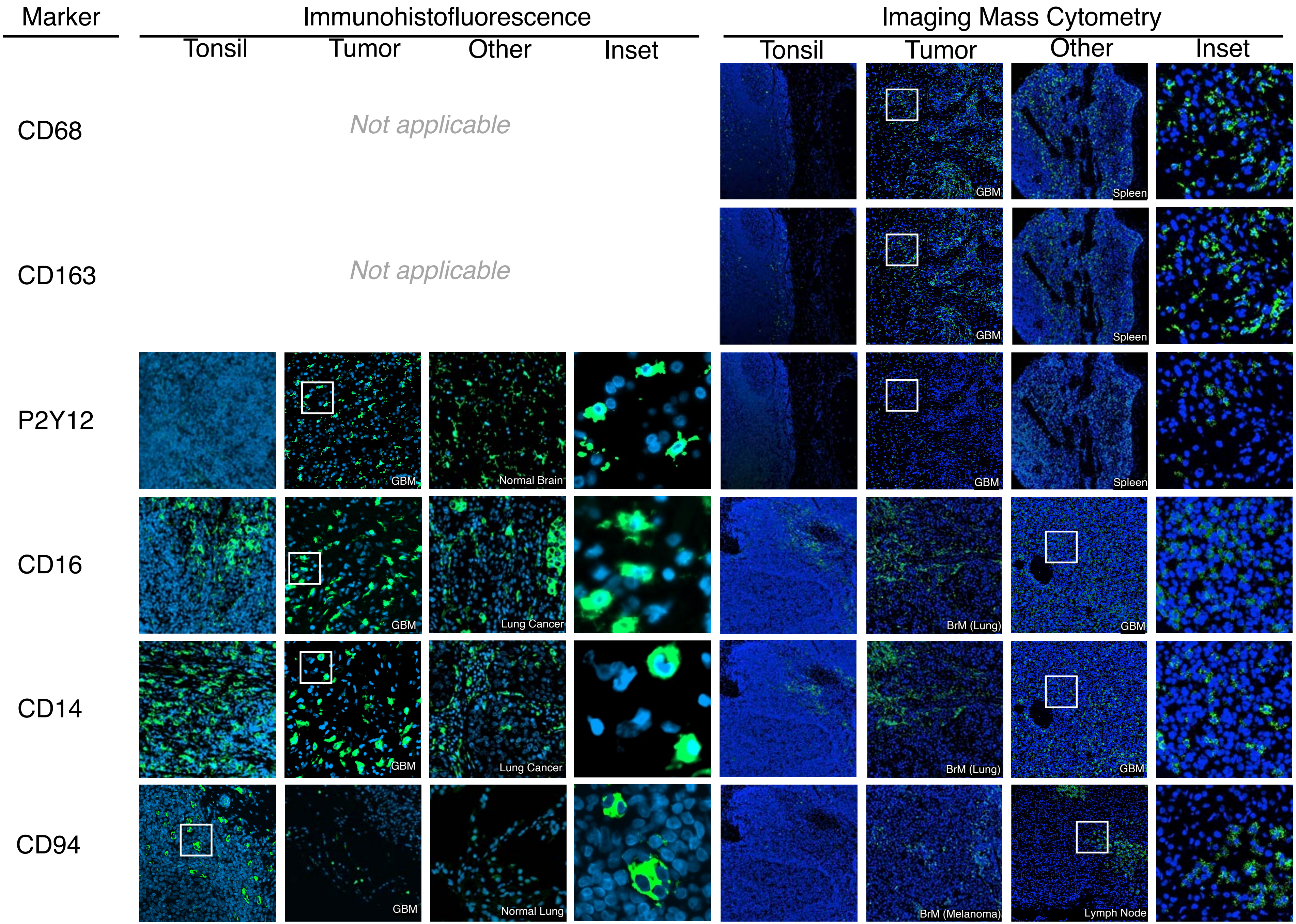

Supplementary Fig. 1 (continued)

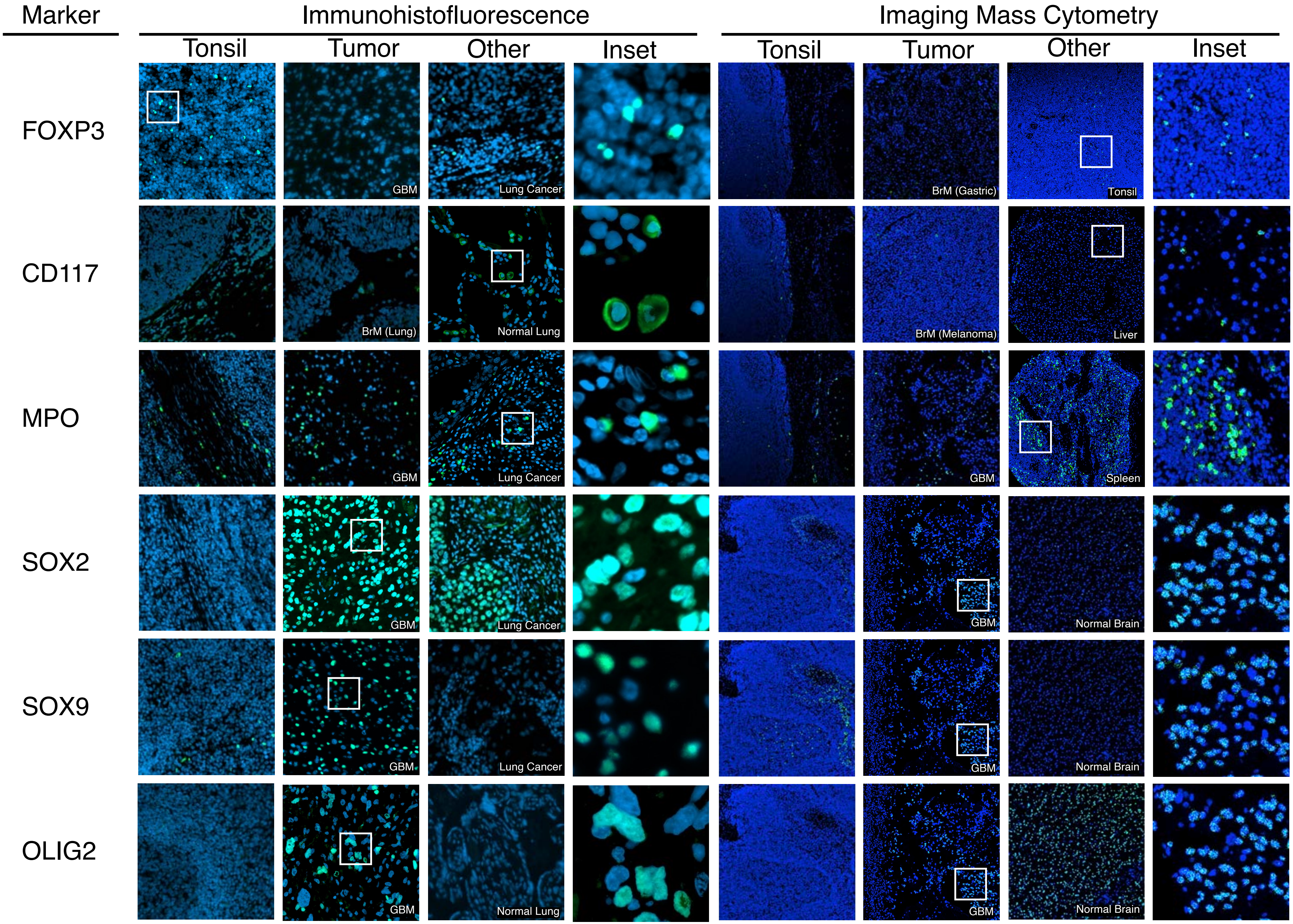

Supplementary Fig. 1 (continued)

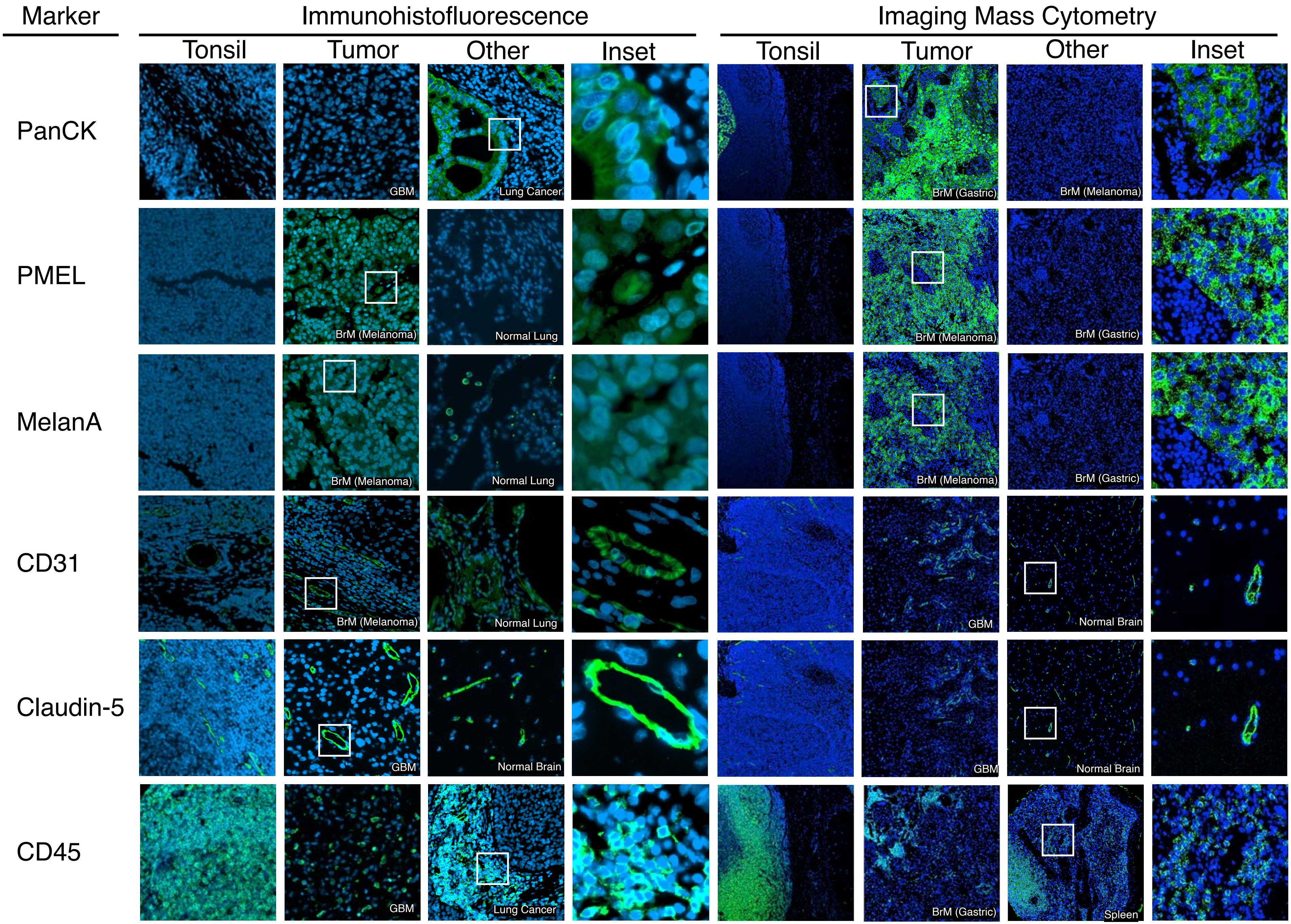

Supplementary Fig. 1 (continued)

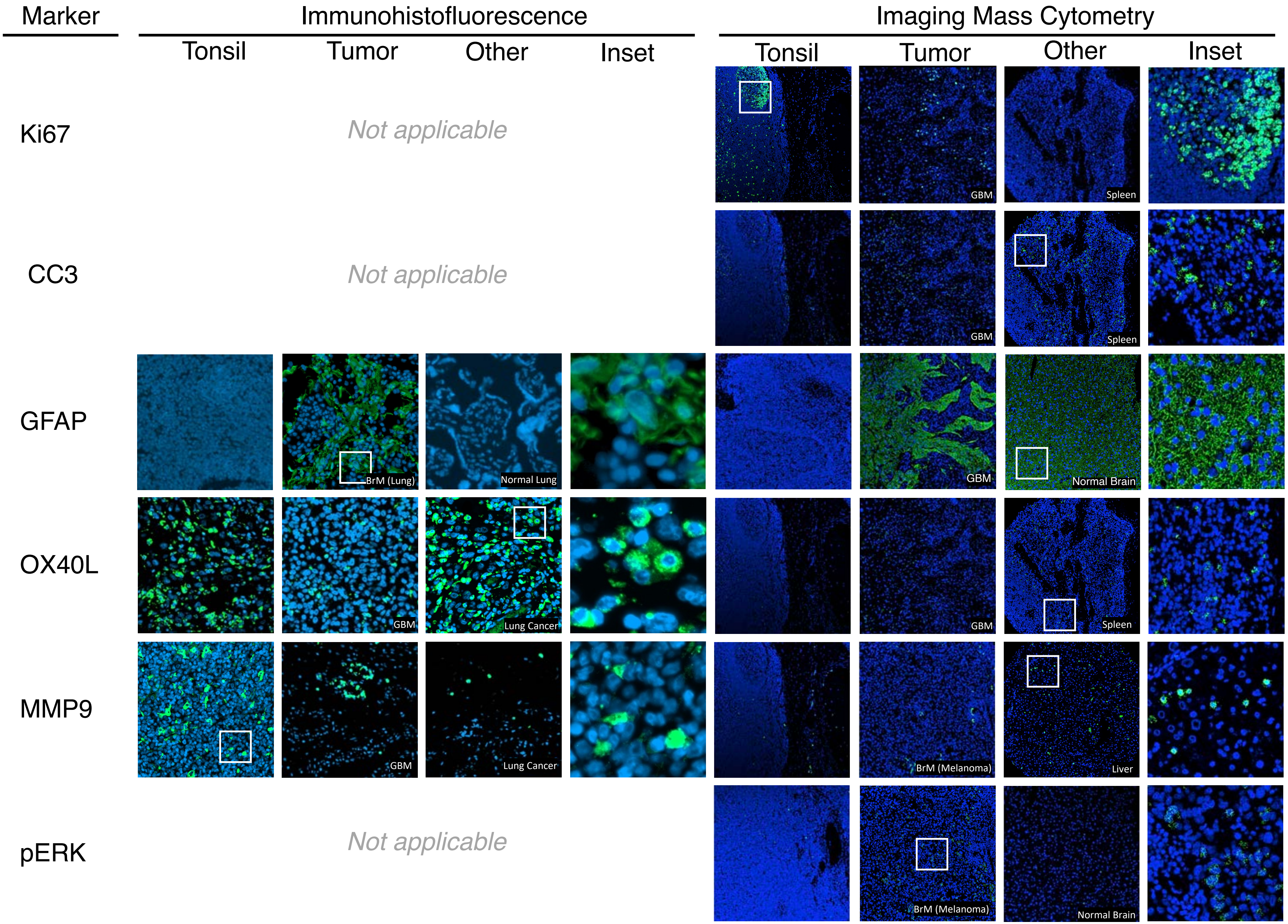

Supplementary Fig. 1 (continued)

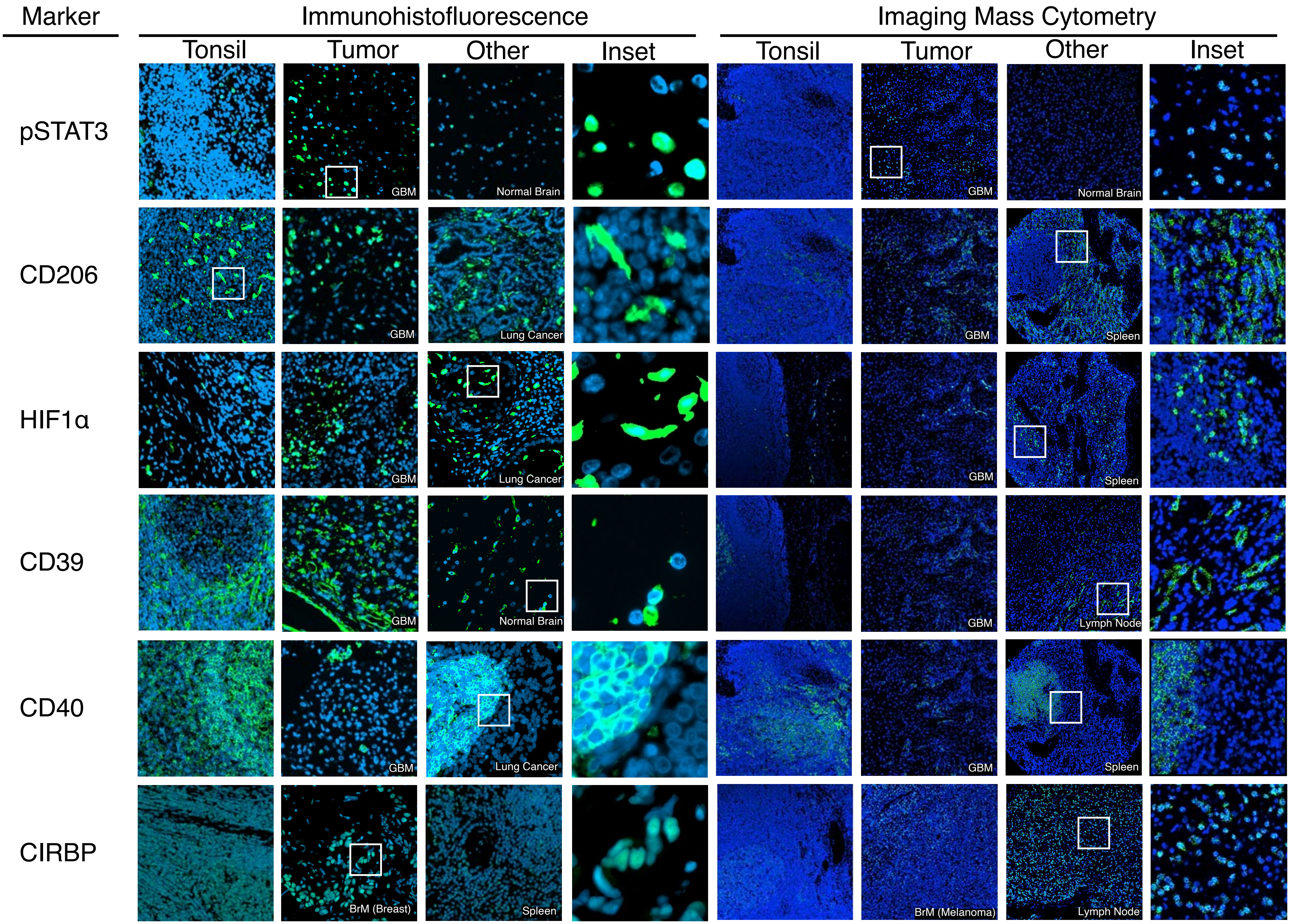

Supplementary Fig. 1 (continued)

**Did not provide consistent staining by IMC; excluded from analysis*

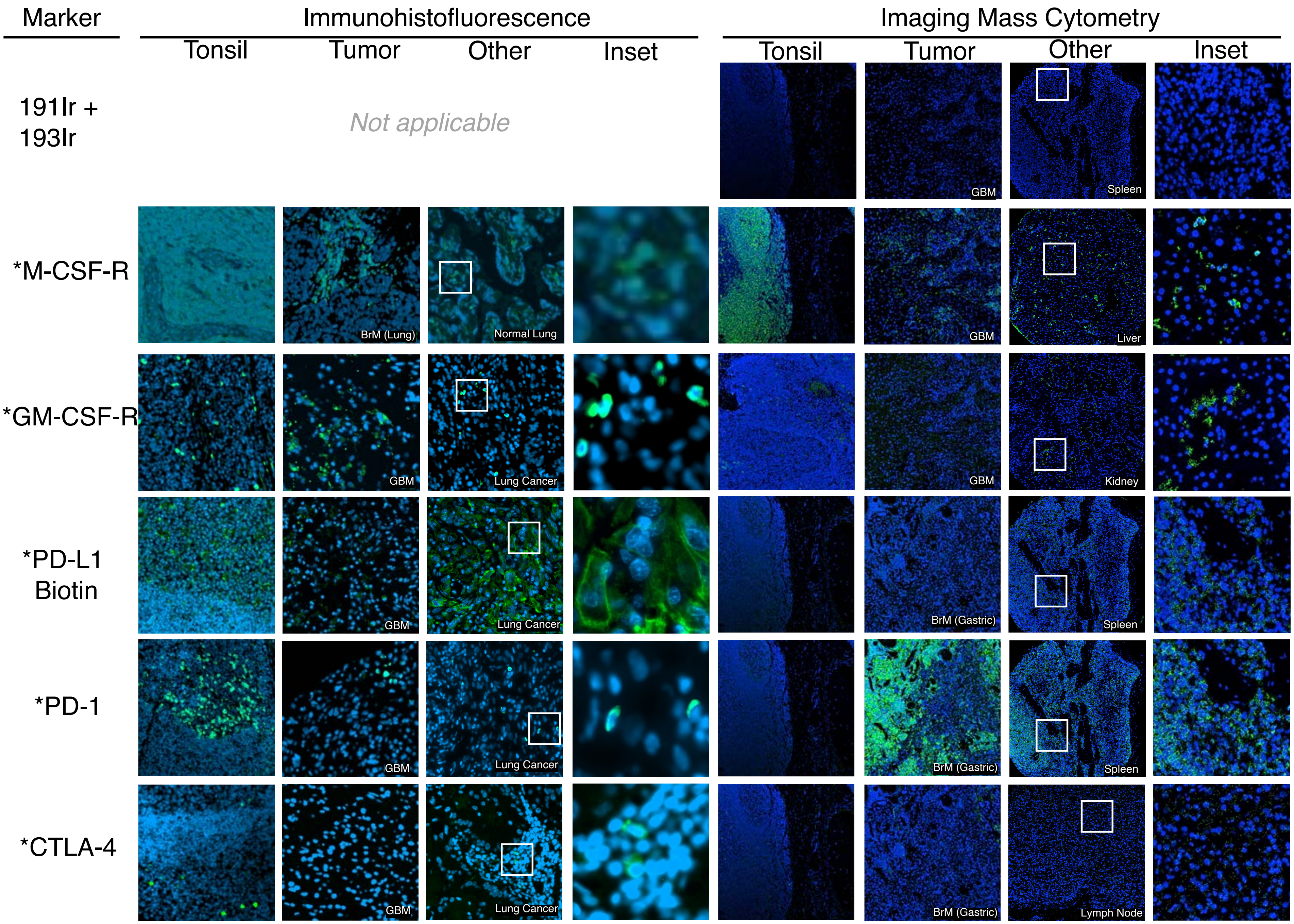

Supplementary Fig. 1: Representative IHF (left) and/or IMC (right) images for all antibodies in the IMC panel. IHF was not performed for pre-conjugated commercial IMC antibodies (with the exception of CD20 and CD45) or Ki67 (clone routinely used). For both applications, control lymphoid tissue (tonsil), tumour (glioblastoma or BrM), and positive or negative control tissue (other) are shown. Images for different antibodies may correspond to different tissue samples. Nuclei (blue) correspond to DAPI or Cell-ID Intercalator-Ir (191Ir or 193Ir). Asterisks denote antibodies with inconsistent IMC staining quality that were excluded from downstream analyses (M-CSF-R, GM-CSF-R, PD-L1, PD-1, CTLA-4).

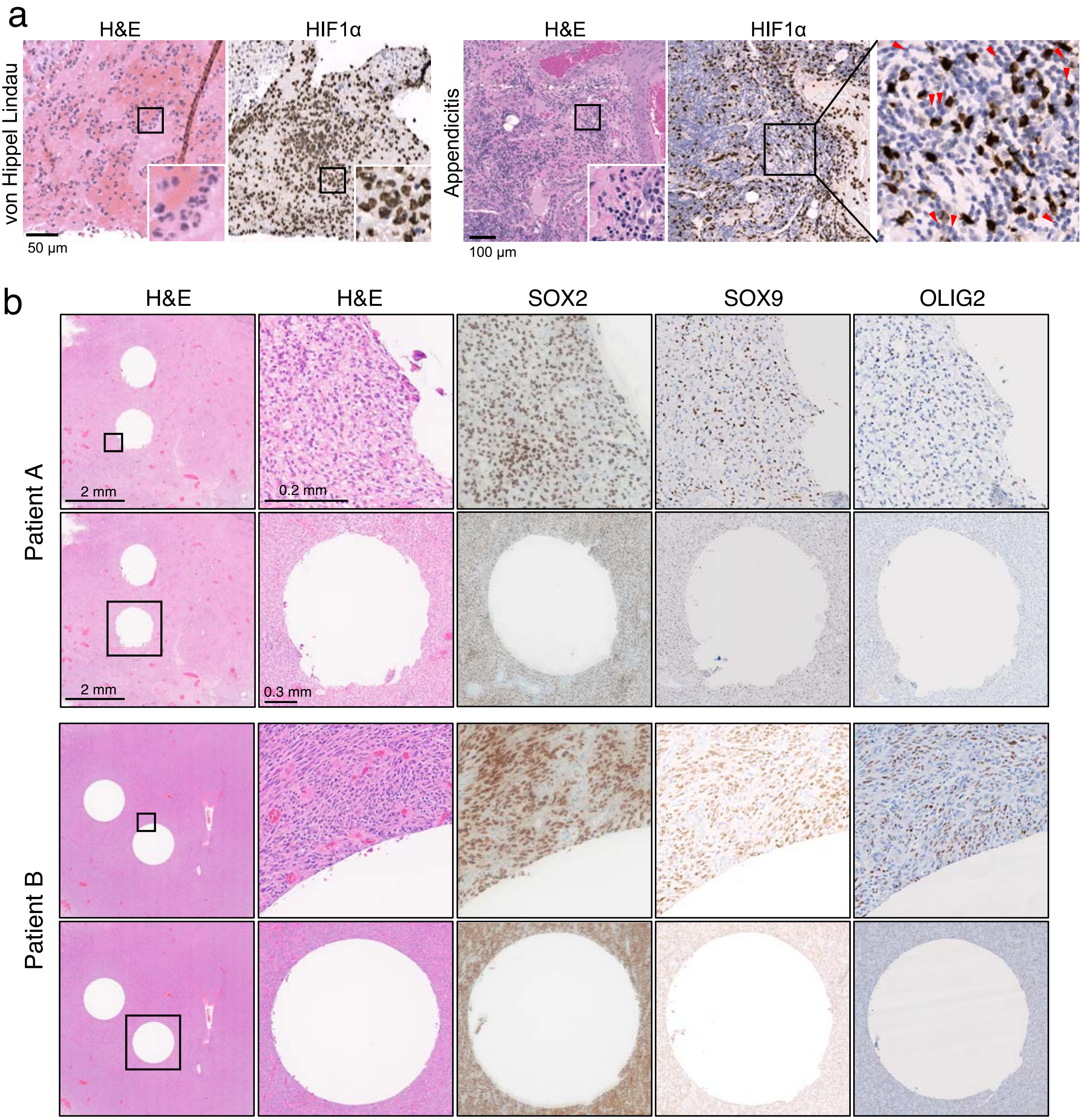

Supplementary Fig. 2: Representative H&E and IHC of HIF1α, SOX2, SOX9 and OLIG2. **a,** HIF1α IHC showing specificity in neutrophils in tissues from patients with von Hippel Lindau syndrome (positive control) and appendicitis (red arrow indicates negative neutrophils). **b,** SOX2, SOX9 and OLIG2 IHC on glioblastoma samples (*n*=5 patient tumors stained per antibody with similar results). The areas sampled for the TMA can be seen (circular white space).

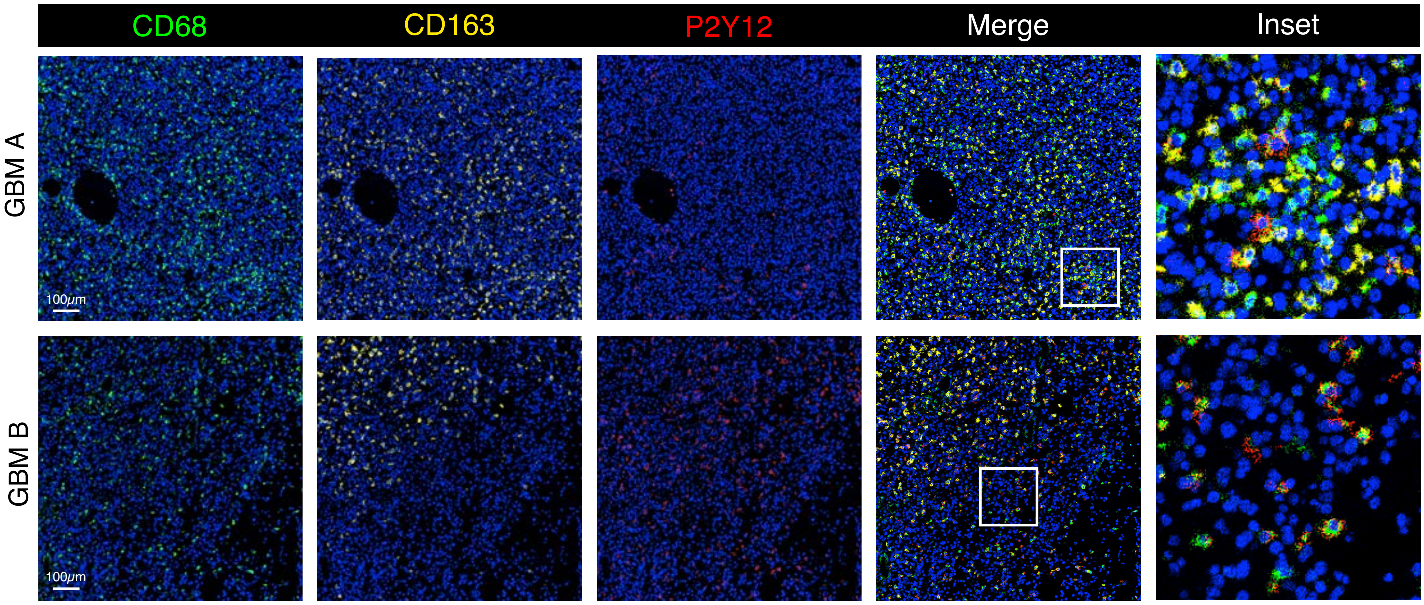

Supplementary Fig. 3: Representative IMC images of macrophage markers. Images show CD68, CD163 P2Y12 in two glioblastoma samples. Blue, DNA (191Ir and 193Ir).

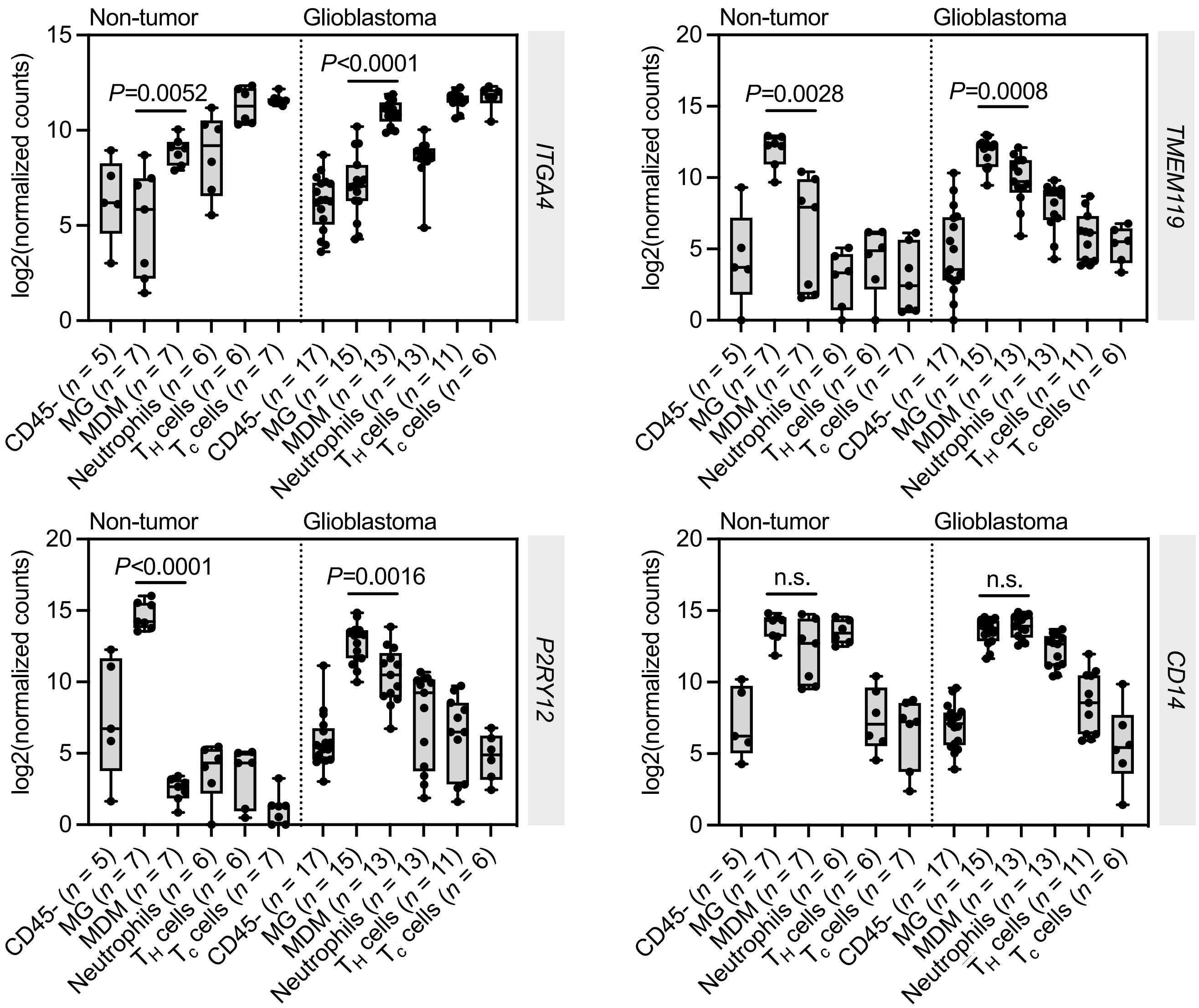

Supplementary Fig. 4: Transcript levels by bulk RNA-seq of canonical macrophage markers across immune cell subsets. P2Y12 (*P2RY12* gene symbol) was used as a microglia marker in our study. Two-sided student's t tests were used for the indicated comparisons between MDM and MG groups (data from ref[16]). Boxes depict IQR, centre line at median, whiskers represent minima and maxima; *n*-values indicate the number of patients.

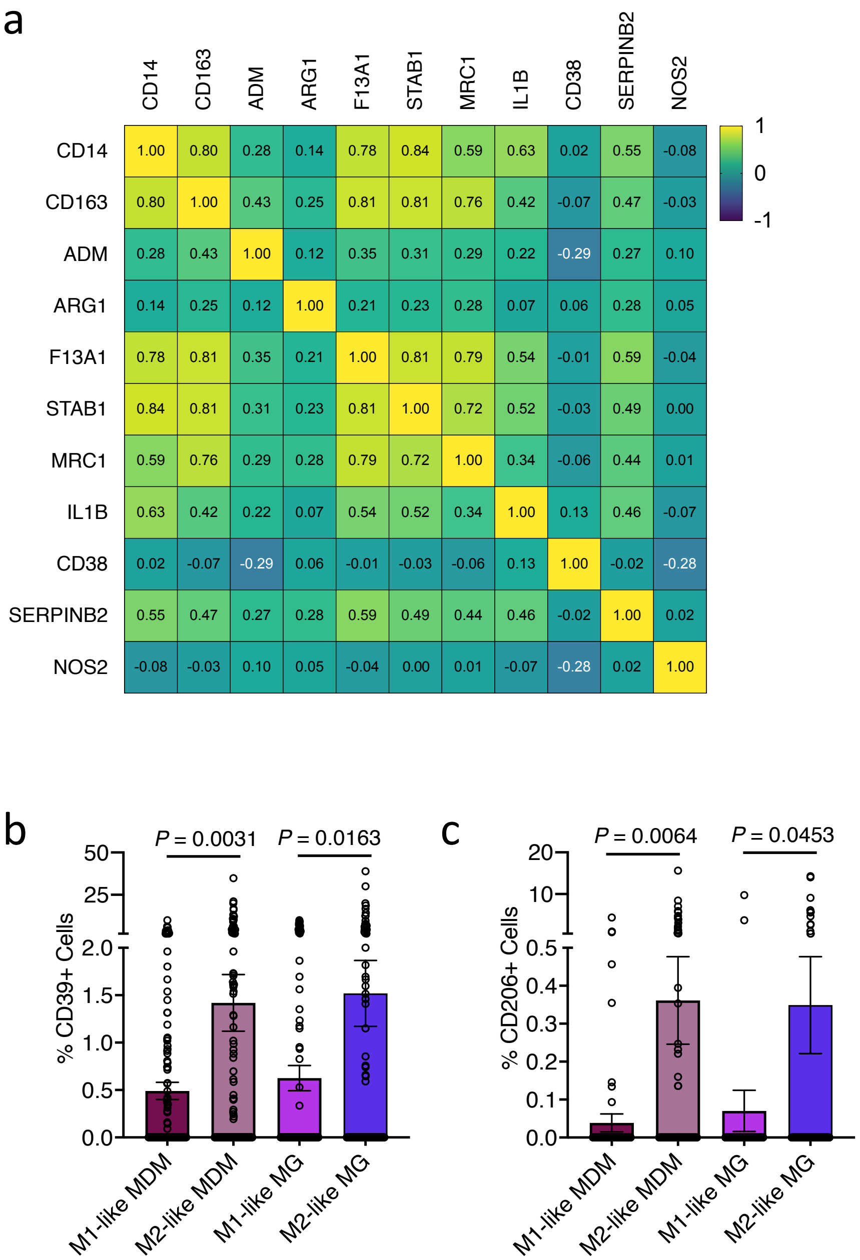

Supplementary Fig. 5: Analysis of M1-like and M2-like macrophage marker expression. a, Spearman correlation of mRNA expression (RNA-seq V2 RSEM) between *CD14* and various M2-like (*CD163, ADM, ARG1, F13A1, STAB1, MRC1*) and M1-like genes (*IL1B, CD38, SERPINB2, NOS2*), demonstrating inconsistent relationships between the expression of these genes in glioblastoma (*n*=166 patients). Data was obtained from The Cancer Genome Atlas (TCGA) and accessed via the cBioPortal for cancer genomics (https://www.cbioportal.org). It should be noted that not all M2-like genes correlate, and similarly not all M1-like genes correlate, exemplifying the complexities of the M1/M2 polarization paradigm outside *in vitro* experimental settings. We chose to use CD163 as a proxy for "M2-like" cells, with these limitations in mind. **b**, Frequency of CD39 expression by IMC in macrophages across glioblastoma tumours (*n*=192 images). Mean ± SEM; Two-sided student's t test. **c**, Frequency of CD206 expression by IMC in macrophages across glioblastoma tumours (*n*=192 images). Mean ± SEM; Two-sided student's t test.

Supplementary Fig. 6

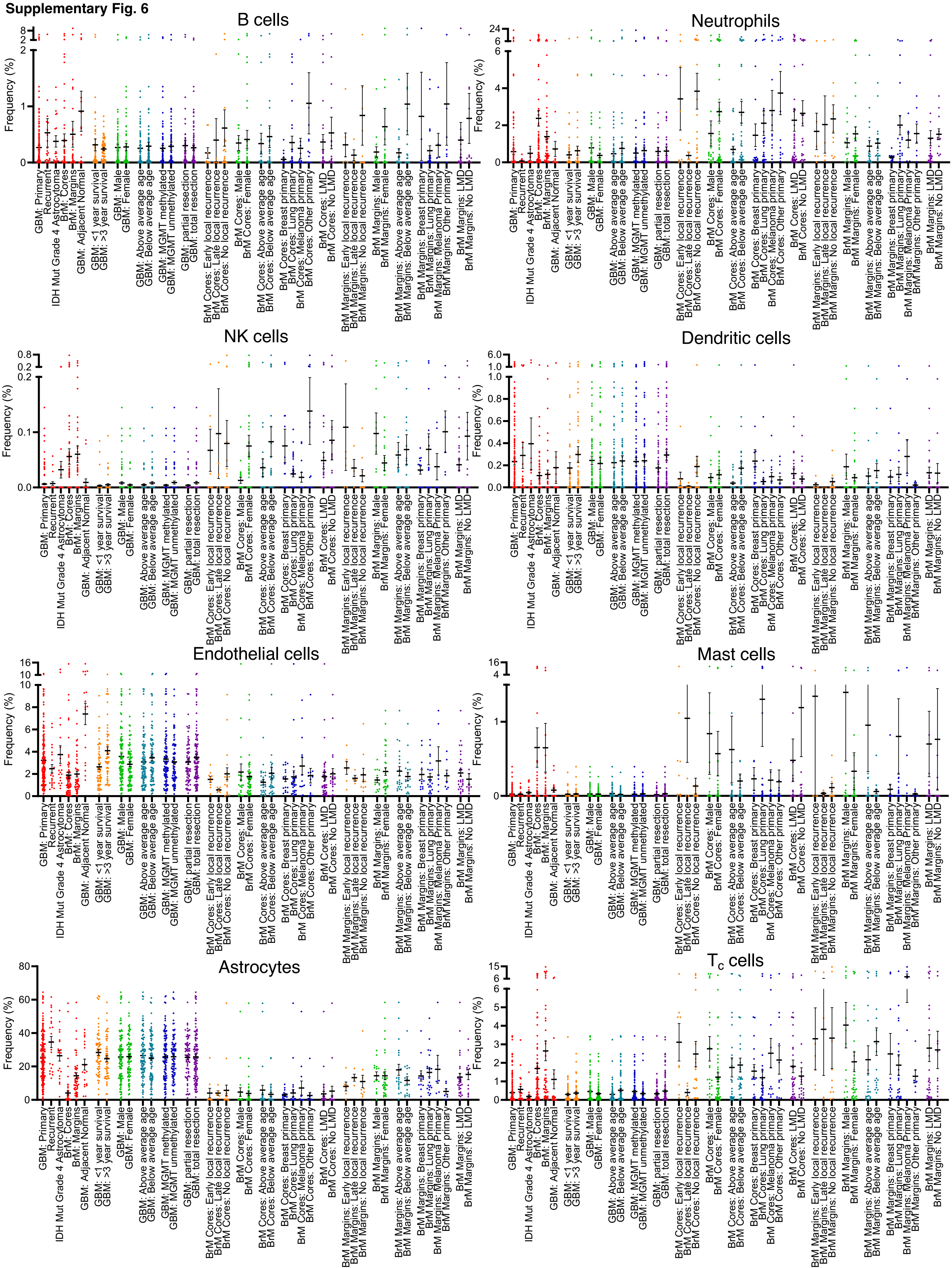

Supplementary Fig. 6 (continued)

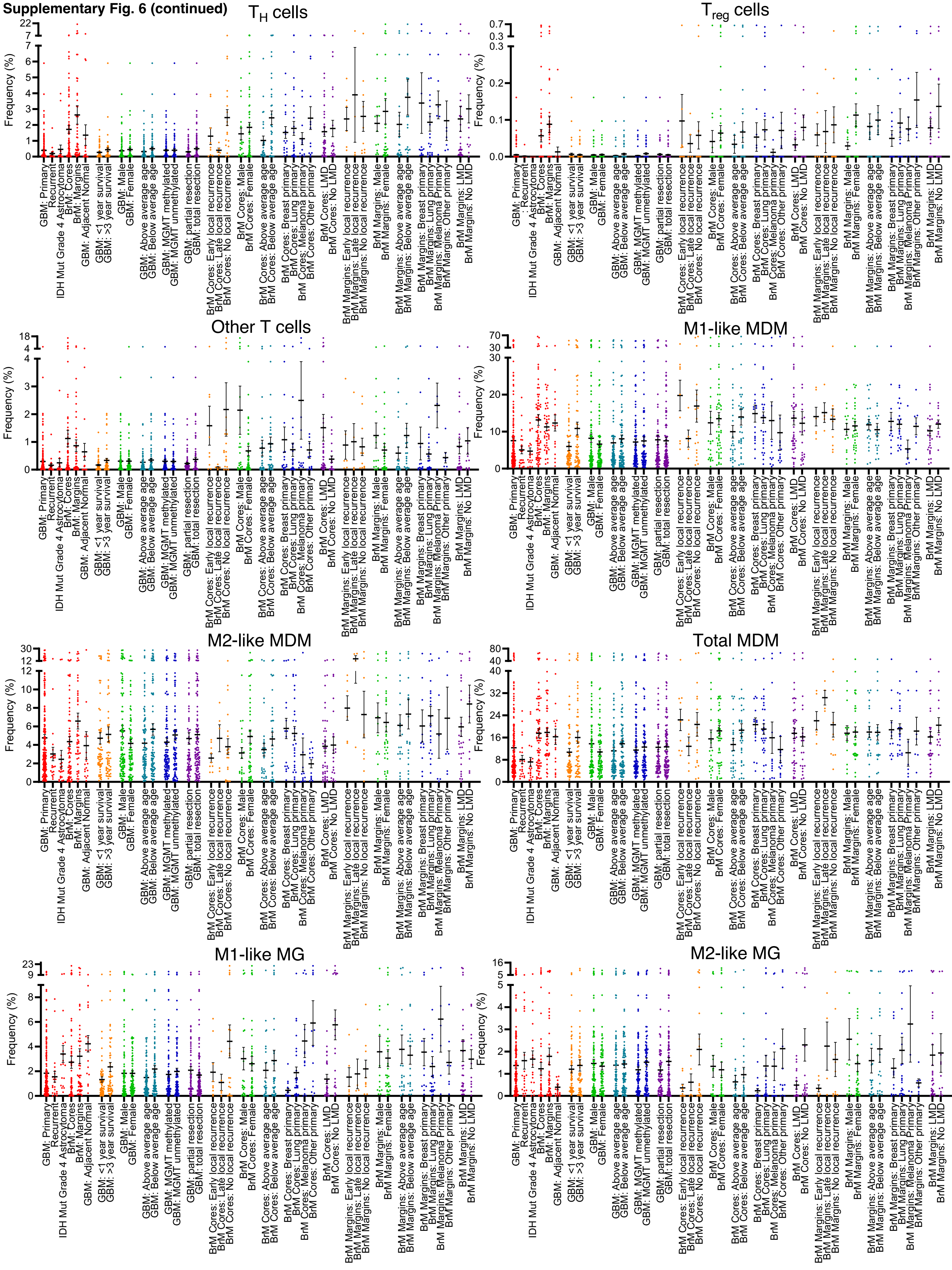

Supplementary Fig. 6 (continued)

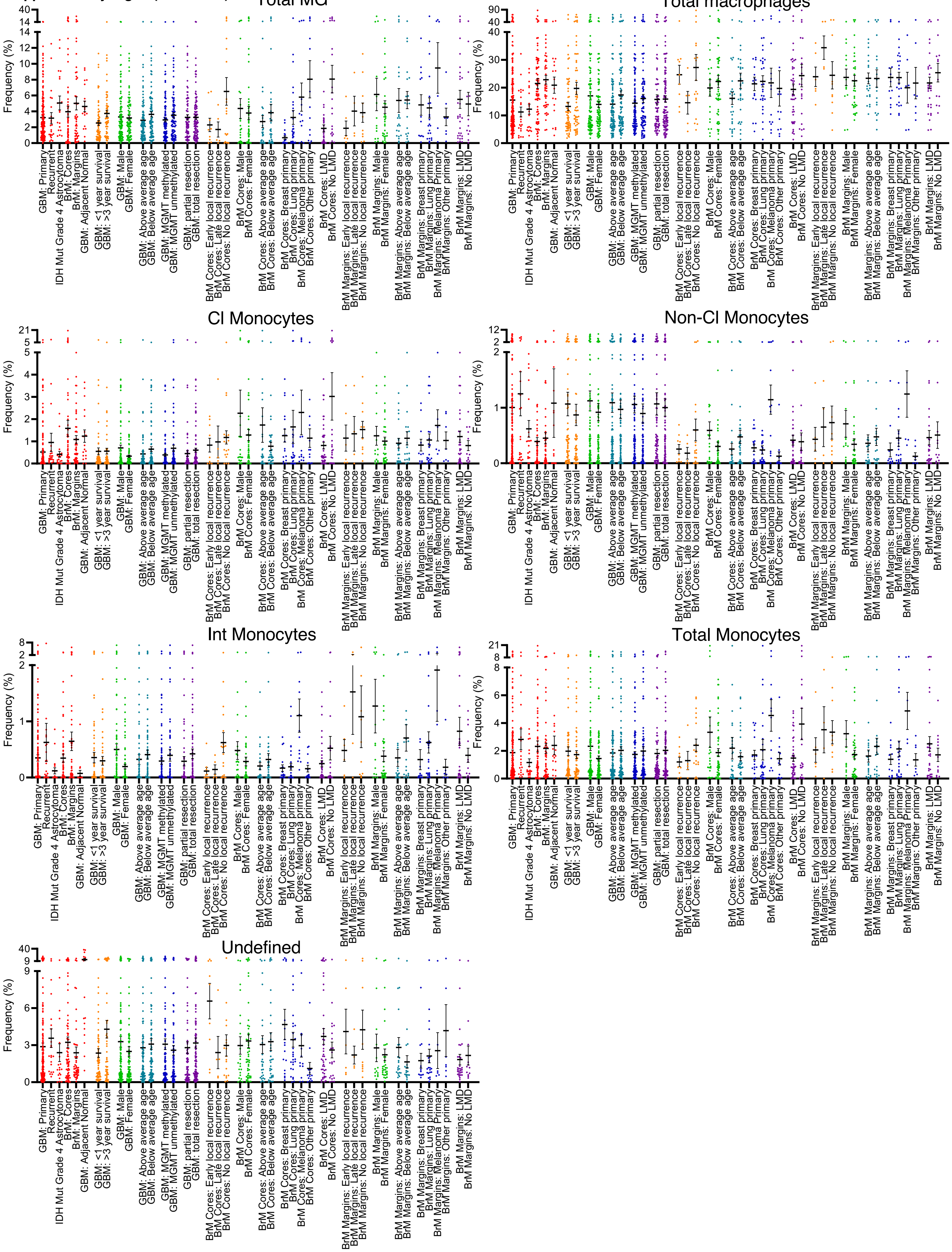

Supplementary Fig. 6: Cell frequencies as a percentage of total cells across clinical subgroups. Mean ± SEM, all datapoints overlaid (1 datapoint/image); statistics represented in Fig. 2a; *n*-values depicted in Fig. 2b.

a

	GBM		BrM-cores		BrM-margins	
	P-Value	Hazard Ratio	P-Value	Hazard Ratio	P-Value	Hazard Ratio
Cancer	0.2826	0.7885	0.2161	0.4074	0.5638	0.6791
Endothelial cells	0.0019	0.4845	0.2156	0.4141	0.1531	2.614
B cells	0.0804	0.6776	0.1905	0.3880	0.1845	2.455
Neutrophils	0.7229	0.9249	0.3194	0.4919	0.5044	0.6378
NK cells	0.2874	0.7469	0.8747	0.8942	0.2211	2.285
Dendritic cells	0.0055	0.5211	0.6486	0.7213	0.3918	0.5501
Mast cells	0.3495	0.8661	0.0313	4.873	0.4078	0.5711
Astrocytes	0.1233	1.406	0.7652	0.8082	0.6223	0.7190
T_c cells	0.4323	0.8417	0.1996	0.3955	0.7560	1.232
T_H cells	0.5065	0.8619	0.0044	0.1231	0.7560	1.232
T_{reg} cells	0.3909	0.8228	0.6992	1.317	0.7384	0.8001
Other T cells	0.2825	0.8288	0.2409	0.4314	0.7884	0.8355
M1-Like MDM	0.3060	0.7944	0.2148	2.452	0.6008	1.430
M2-Like MDM	0.8163	1.052	0.1690	0.3658	0.3420	1.887
M1-Like MG	0.9616	0.9893	0.5176	0.6310	0.0093	0.1634
M2-Like MG	0.7683	1.067	0.2999	0.4751	0.3504	0.5283
Cl Mono	0.7912	0.9436	0.0586	0.2510	0.3780	0.5489
Non-Cl Mono	0.5504	0.8759	0.2475	2.346	0.4433	1.673
Int Mono	0.1585	0.7288	0.0343	0.2120	0.2137	2.317
Undefined	0.8123	0.9495	0.0749	3.590	0.6015	1.496

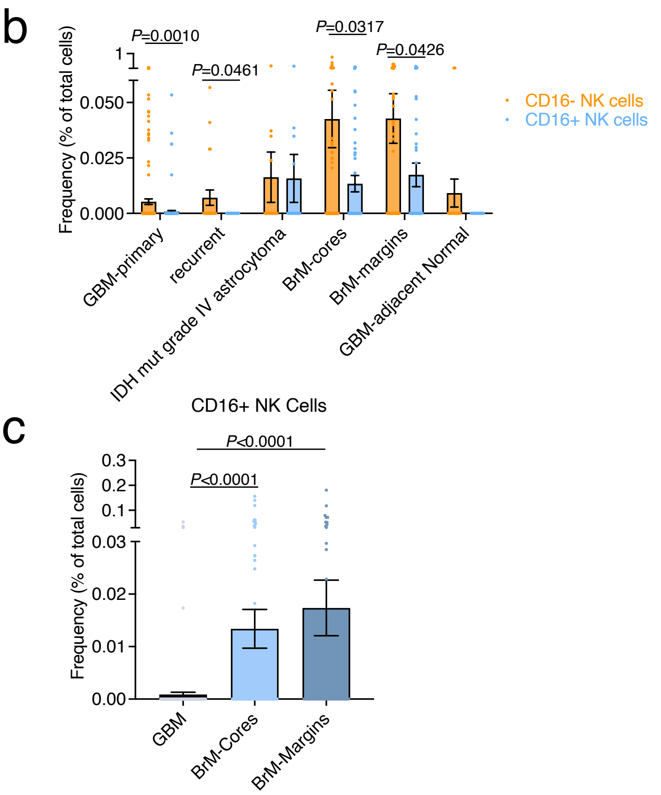

Supplementary Fig. 7: Cell survival associations and CD16+ NK cell dynamics. a, Log rank (Mantel-Cox) *P*-values and hazard ratios obtained from Kaplan-Meier analysis of glioblastoma (*n*=86 patients), BrM-cores (*n*=26 patients) and BrM-margins (*n*=27 patients). Patients were ranked based on cell frequencies as a percentage of total cells and assigned to high vs low groups based on median. Significant survival associations are highlighted in green (decreased risk) and red (increased risk). Cell frequencies were averaged when multiple images corresponded to the same patient. For data sets with an odd number of patients, an extra patient was consistently assigned to either the high or low group. For cell types where >50% of patients had zero cells (i.e. more than the median value), all patients with zero cells were assigned to the low group. **b**, Frequency of CD16+ and CD16- NK cells as a percentage of total cells in primary glioblastoma (*n*=192 images), recurrent (*n*=22 images), IDH mutant grade IV astrocytoma (*n*=19 images), BrM-cores (*n*=72 images), BrM-margins (*n*=47 images) and glioma-adjacent normal tissue (*n*=18 images) Graph depicts mean ± SEM; Two-sided student's t test. **c**, A direct comparison of CD16+ NK cells in glioblastoma (*n*=192 images), BrM-cores (*n*=72 images), and BrM-margins (*n*=47 images). Graph depicts mean ± SEM; One-way ANOVA.

Supplementary Fig. 8

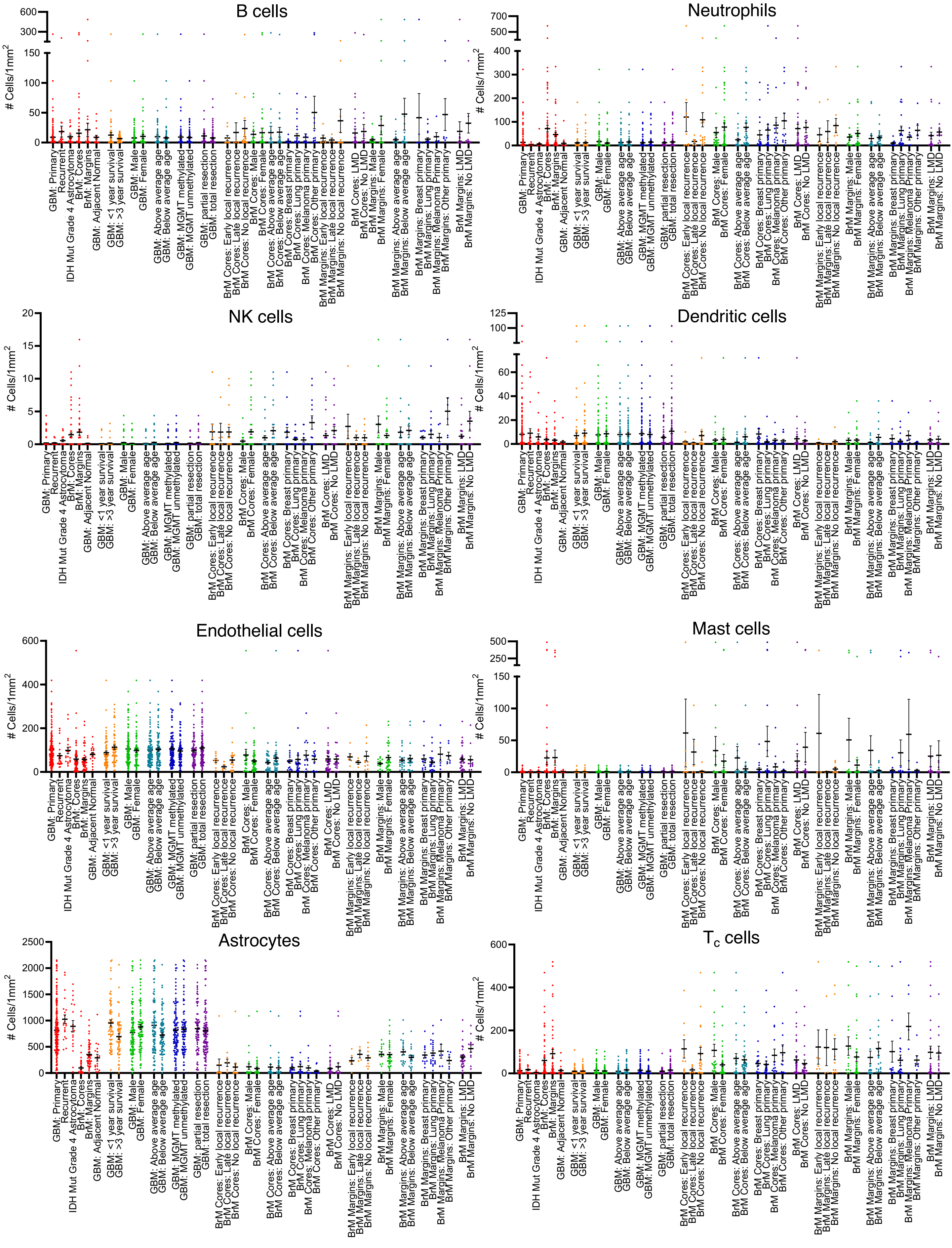

Supplementary Fig. 8 (continued)

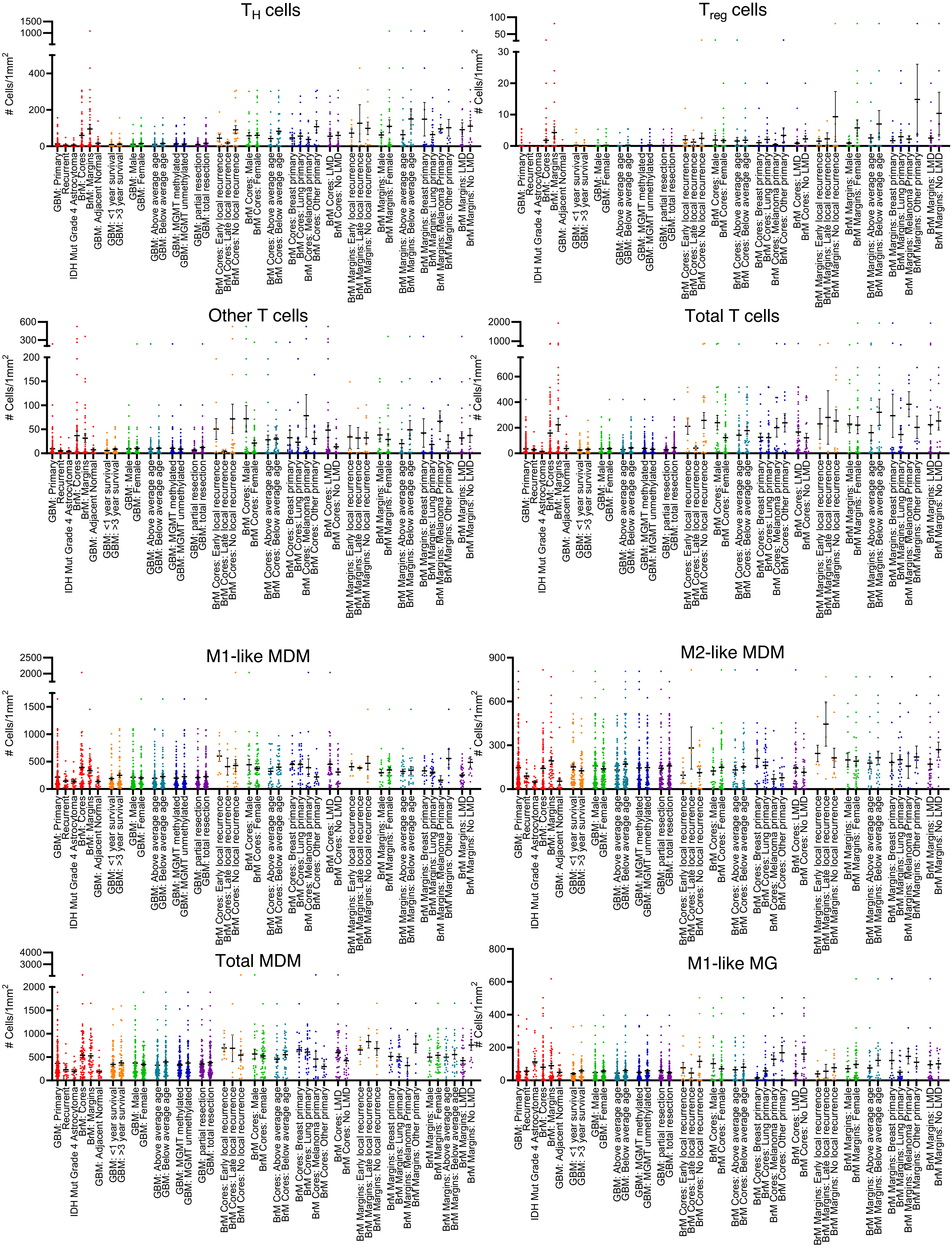

Supplementary Fig. 8 (continued)

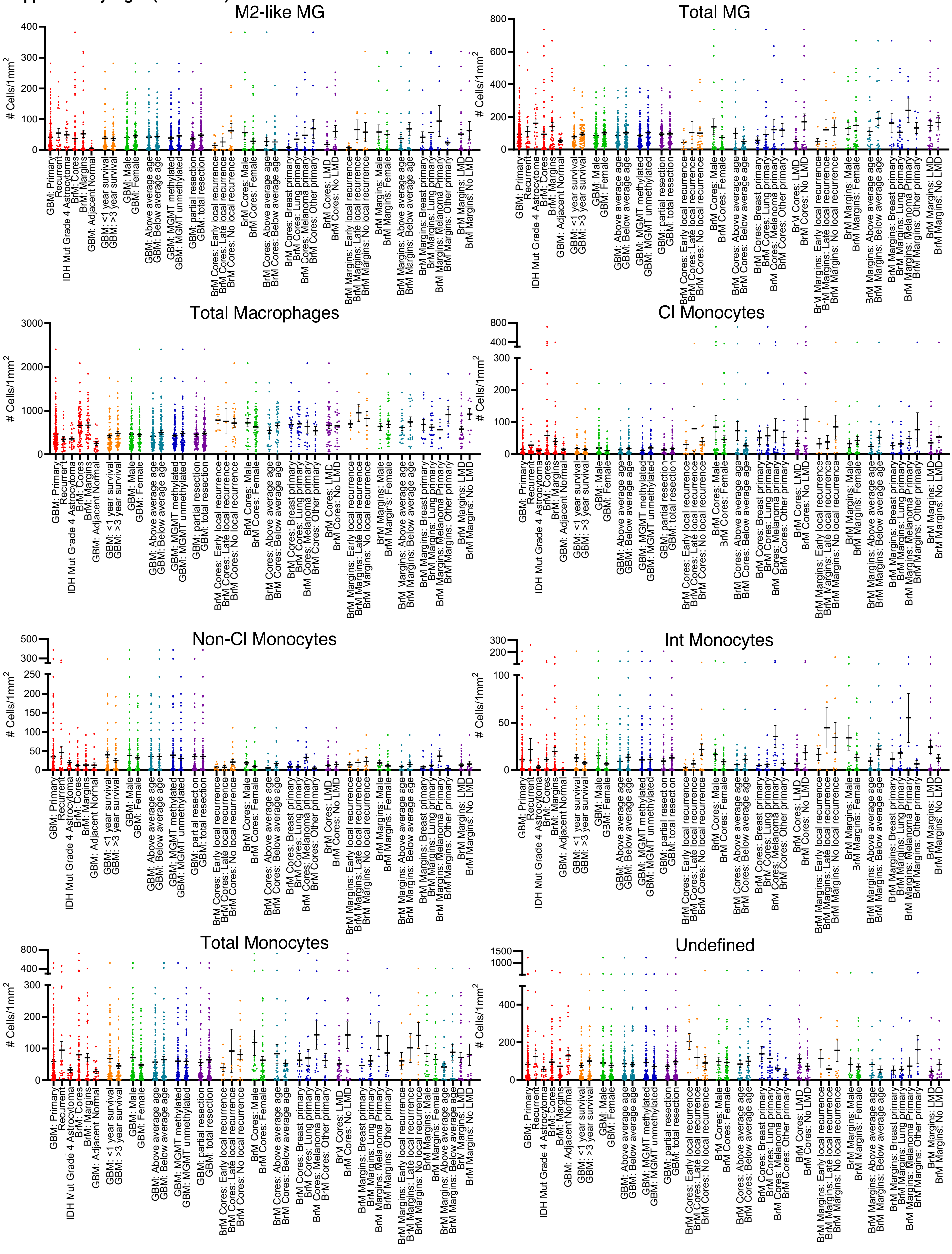

Supplementary Fig. 8: Cell density (number of cells/mm^2) for each cell type across clinical subgroups. Mean ± SEM, all datapoints overlaid (1 datapoint/image); statistics represented in Extended Data Fig. 4a*; n*-values depicted in Fig. 2b.

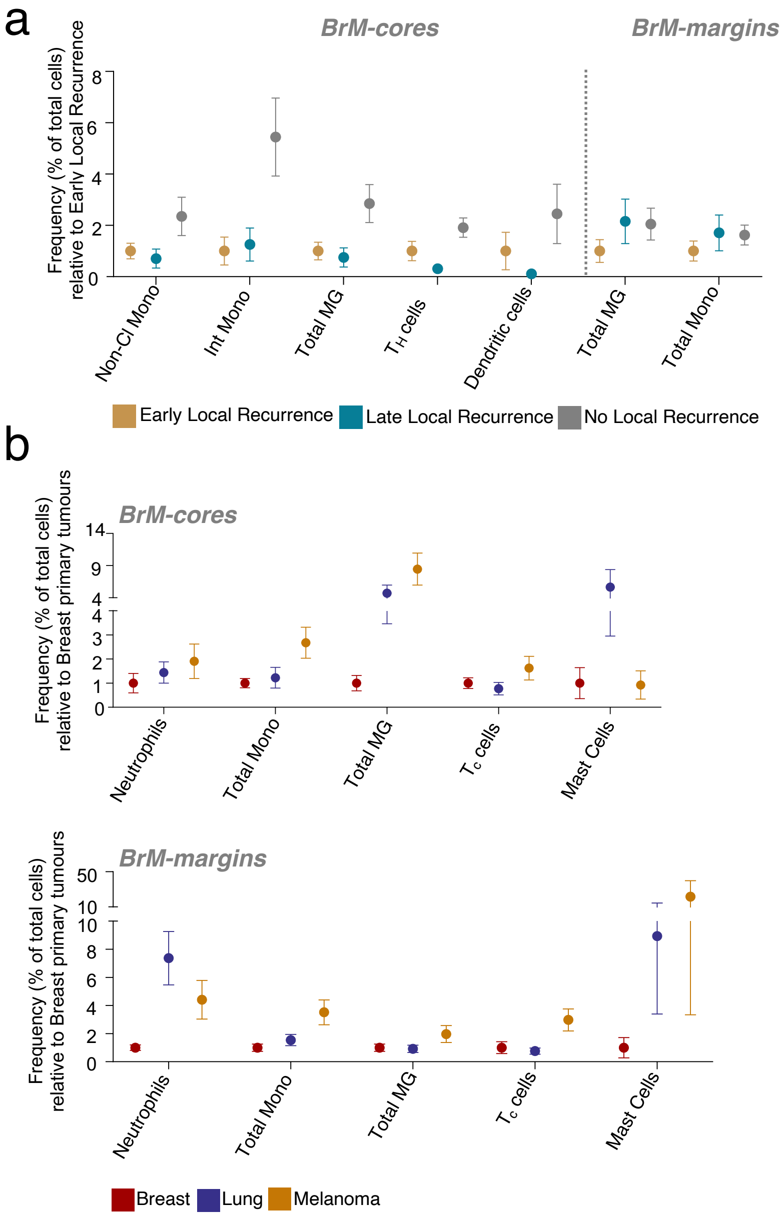

Supplementary Fig. 9: Comparison of cell frequencies in BrM patients based on local recurrence or primary tumour. a, Average frequency of cell types in patients with early (*n*=9 core, *n*=6 margin images), late (*n*=5 core, *n*=4 margin images) or no local recurrence (*n*=19 core, *n*=10 margin images) across BrM-cores and BrM-margins. Graph depicts frequencies relative to early local recurrence ± SEM. **b**, Average frequency of cell types across BrM-cores (top) and BrM-margins (bottom) arising from breast (*n*=17 core, *n*=12 margin images), lung (*n*=29 core, *n*=22 margin images) or melanoma (*n*=13 core, *n*=6 margin images) primary tumours. Graph depicts frequencies relative to breast BrM ± SEM. All data relate to Supplementary Fig. 6; statistics in Fig. 2a.

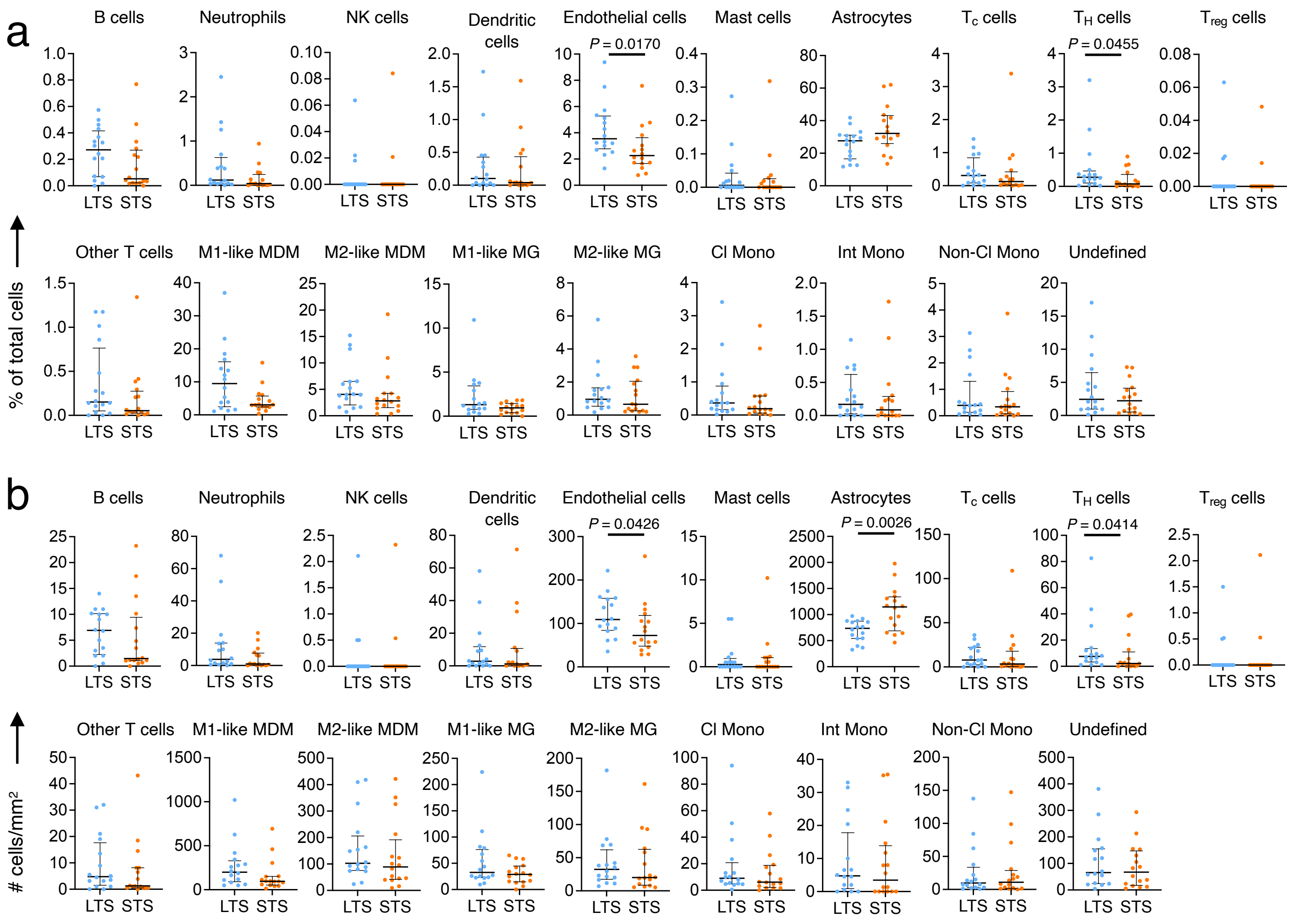

Supplementary Fig. 10: Comparison of cell types in glioblastoma LTS versus STS. a, Cell frequencies as a percentage of total cells in the glioblastoma LTS/STS cohort. Cell frequencies were averaged when multiple samples corresponded to the same patient. Median ± IQR; Two-sided Mann-Whitney test; *n*=16 patients/group. **b**, Cellular density (number of cells/mm^2) for each cell type in the glioblastoma LTS/STS cohort. Cellular density was averaged when multiple samples corresponded to the same patient. Median ± IQR; Two-sided Mann-Whitney test, *n*=16 patients/group.

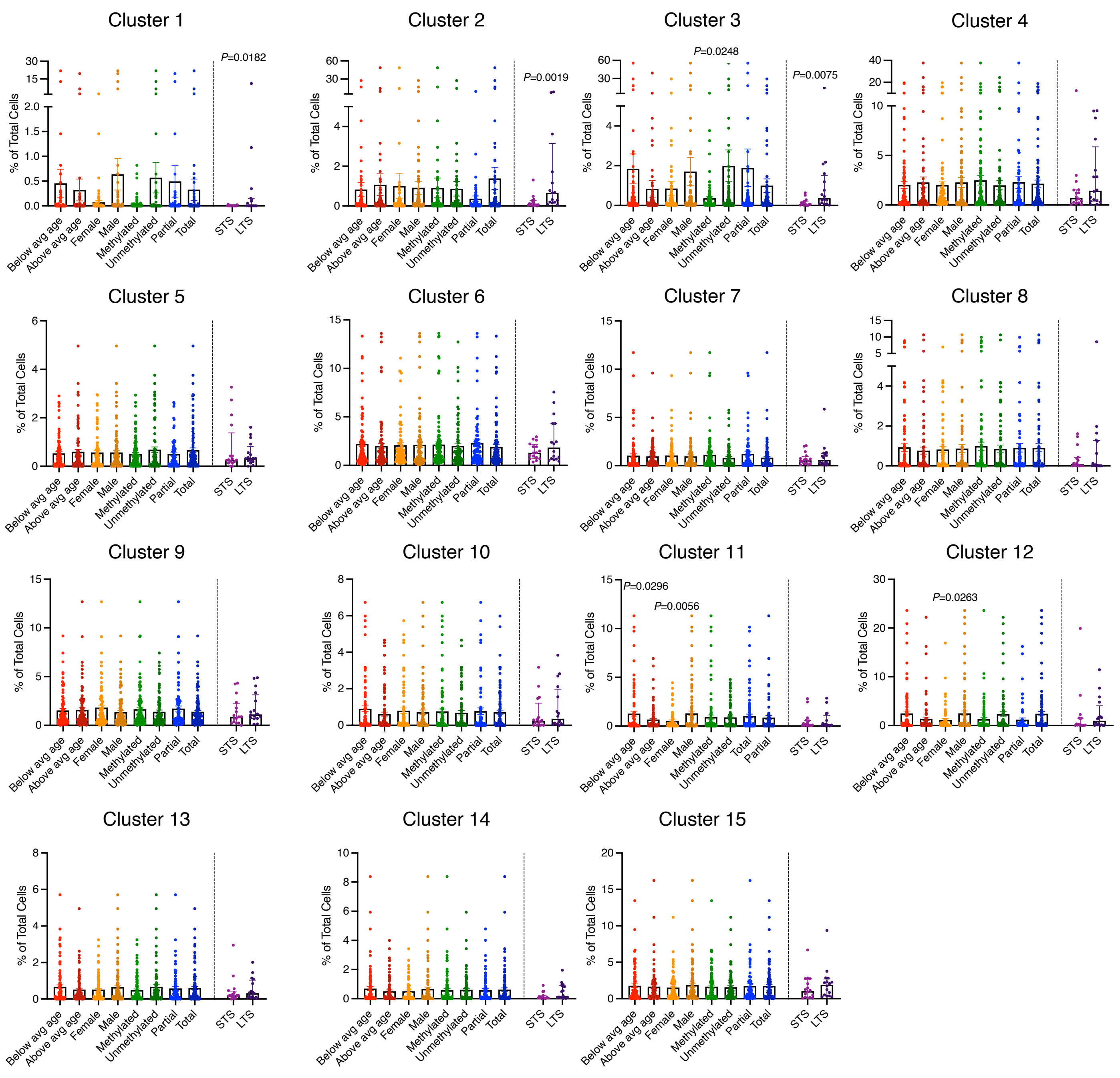

Supplementary Fig. 11: Comparison of macrophage/monocyte clusters across glioblastoma clinical groups. Frequency of clusters 1-15 corresponding to Fig. 3f-h across glioblastoma clinical groups (*n* =192 images) and the LTS/STS cohort (*n*=32 patients). For clinical groups: Mean ± SEM; Two-sided student's t test. For LTS/STS cohort: Median ± IQR; Two-sided Mann-Whitney test.

Supplementary Table 1: Antibody panel information for imaging mass cytometry.

	Target	Samples Used On	Metal Conjugate	Clone	Dilution	Catolog #	Supplier	
Lymphoid lineage	CD3	GBM, BrM	170Er	Polyclonal	1:50	3170019D	Fluidigm	
	CD8a	GBM, BrM	162Dy	C8/144	1:50	3162034D	Fluidigm	
	CD4	GBM, BrM	156Gd	EPR6855	1:100	ab133616	Abcam	
	FOXP3	GBM, BrM	155Gd	236A/E7	1:50	ab20034	Abcam	
	CD20	GBM, BrM	161Dy	H1	1:100	3161029D	Fluidigm	
	CD94	GBM, BrM	166Er	EPR21003	1:100	ab235441	Abcam	
Myeloid Lineage	CD68	GBM, BrM	159Tb	KP1	1:50	3159035D	Fluidigm	
	CD163	GBM, BrM	147Sm	EDHu-1	1:200	3147021D	Fluidigm	
	P2Y12	GBM, BrM	169Tm	Polyclonal	1:100	AS-55043A	Labscoop	
	CD11c	GBM, BrM	154Sm	EP1347Y	1:100	ab52632	Abcam	
	HLA-DR	GBM, BrM	174Yb	EPR3692	1:100	ab92511	Abcam	
	CD14	GBM, BrM	144Nd	SP192	1:100	ab183322	Abcam	
	CD16	GBM, BrM	142Nd	SP175	1:100	ab183354	Abcam	
	CD117	GBM, BrM	153Eu	YR145	1:100	ab32363	Abcam	
	MPO	GBM, BrM	145Nd	EPR20257	1:100	ab208670	Abcam	
Compartment Markers	PanCK	BrM only	175Lu	AE1+AE3	1:100	ab80826	Abcam	
	PMEL	BrM only	165Ho	HMB-45	1:100	NBP2-34638	Novus Biologicals	
	MelanA	BrM only	168Er	A103	1:100	sc-20032	Santa Cruz	
	SOX2	GBM only	175Lu	EPR3131	1:200	ab215970	Abcam	
	SOX9	GBM only	146Nd	EPR14335-78	1:100	ab185966	Abcam	
	OLIG2	GBM only	171Yb	EPR2673	1:200	ab220796	Abcam	
	CD31	GBM, BrM	176Yb	JC/70A	1:100	ab9498	Abcam	
	GFAP	GBM, BrM	141Pr	EP672Y	1:400	ab33922	Abcam	
	CD45	GBM, BrM	152Sm	D9M8I	1:100	3152018D	Fluidigm	
Functional Markers	Ki67	GBM, BrM	143Nd	B56	1:100	ab279657	Abcam	
	CC3	GBM, BrM	172Yb	5A1E	1:100	3172027D	Fluidigm	
	Claudin-5	GBM, BrM	173Yb	EPR7583	1:100	ab131259	Abcam	
	OX40L	GBM, BrM	164Dy	EP1168Y	1:100	ab76130	Abcam	
	MMP9	GBM, BrM	160Gd	EP1255Y	1:100	ab137867	Abcam	
	M-CSF-R	GBM, BrM	151Eu	SP211	1:100	ab183316	Abcam	*
	GM-CSF-R	GBM only	165Ho	4H1	1:50	305902	Biolegend	*
	CTLA-4	BrM only	158Gd	SP355	1:100	ab227709	Abcam	*
	HIF1α	GBM, BrM	149Sm	EP1215Y	1:100	ab51608	Abcam	
	CD39	GBM, BrM	167Er	EPR20627	1:100	ab223842	Abcam	
	CD40	GBM only	168Er	EPR20540	1:50	ab213205	Abcam	
	pERK	BrM only	171Yb	D13.14.4E	1:100	3171010A	Fluidigm	
	pSTAT3	GBM, BrM	163Dy	EP2147Y	1:100	ab76315	Abcam	
	CIRBP	BrM only	146Nd	EPR18783	1:100	ab238946	Abcam	
	CD206	GBM only	158Gd	EPR22489-7	1:100	ab254471	Abcam	
	PD-L1	GBM, BrM	Biotin	E1L3N	1:50	13684T	Cell Signaling Technologies	*
	Biotin		150Nd	1D4-C5	1:50	3150008B	Fluidigm	
	PD-1	GBM, BrM	148Nd	D4W2J	1:50	86163	Cell Signaling Technologies	*

*Antibodies did not provide consistent staining by IMC; excluded from analysis

Supplementary Table 2: Clinical characteristics of the short-term survivor (STS) and long-term survivor (LTS) patient cohorts.

	STS	LTS	Two-Sided Fisher's Exact Test (P<0.1)
Partial Resection	0	0	ns
Total Resection	16	16	ns
MGMT methylated	6	11	ns
MGMT unmethylated	10	5	ns
Age < 50	2	3	ns
Age ≥ 50	14	13	ns
Male	5	6	ns
Female	11	10	ns
