# Supplementary material for: Single-cell spatial immune landscapes of primary and metastatic brain tumours
Source: Nature. 2023 Feb 1;614(7948):555–63. doi: 10.1038/s41586-022-05680-3 (PMC9931580; doi:10.1038/s41586-022-05680-3)
Supplement: Supplementary file 2 — Reporting Summary [file 41586_2022_5680_MOESM2_ESM.pdf]

nature portfolio

Corresponding author(s): Peter M. Siegel, Logan A. Walsh, Daniela F. Quail

Last updated by author(s): Dec 2, 2022

Reporting Summary

Nature Portfolio wishes to improve the reproducibility of the work that we publish. This form provides structure for consistency and transparency in reporting. For further information on Nature Portfolio policies, see our Editorial Policies and the Editorial Policy Checklist.

Statistics

For all statistical analyses, confirm that the following items are present in the figure legend, table legend, main text, or Methods section.

n/a	Confirmed	
☐	☒	The exact sample size (*n*) for each experimental group/condition, given as a discrete number and unit of measurement
☐	☒	A statement on whether measurements were taken from distinct samples or whether the same sample was measured repeatedly
☐	☒	The statistical test(s) used AND whether they are one- or two-sided<br>*Only common tests should be described solely by name; describe more complex techniques in the Methods section.*
☒	☐	A description of all covariates tested
☐	☒	A description of any assumptions or corrections, such as tests of normality and adjustment for multiple comparisons
☐	☒	A full description of the statistical parameters including central tendency (e.g. means) or other basic estimates (e.g. regression coefficient) AND variation (e.g. standard deviation) or associated estimates of uncertainty (e.g. confidence intervals)
☐	☒	For null hypothesis testing, the test statistic (e.g. *F*, *t*, *r*) with confidence intervals, effect sizes, degrees of freedom and *P* value noted<br>*Give P values as exact values whenever suitable.*
☒	☐	For Bayesian analysis, information on the choice of priors and Markov chain Monte Carlo settings
☒	☐	For hierarchical and complex designs, identification of the appropriate level for tests and full reporting of outcomes
☐	☒	Estimates of effect sizes (e.g. Cohen's *d*, Pearson's *r*), indicating how they were calculated

Our web collection on statistics for biologists contains articles on many of the points above.

Software and code

Policy information about availability of computer code

Data collection	Data collection for imaging mass cytometry was performed using the Hyperion Imaging System CyTOF Software version 6.7.1014.
Data analysis	Code for IMC cell segmentation was written in Matlab (version 2019b) and Python (version 3.7.12). All code, libraries and algorithms (with versions) are deposited on GitHub with detailed descriptions and can be accessed at: https://github.com/walsh-quail-labs/IMC-Brain<br>Prism 9.1.0 (GraphPad) and RStudio (version 4.2.0) were used for statistical analyses. Immunohistofluorescent image analysis was performed in HALO (version 3.5). Pathway enrichment analyses were performed with IPA Software (version 01-13). Area analysis of IMC images was performed using ImageJ (1.53k). scRNA-seq data was analyzed using Seurat (version 4.1.1).

For manuscripts utilizing custom algorithms or software that are central to the research but not yet described in published literature, software must be made available to editors and reviewers. We strongly encourage code deposition in a community repository (e.g. GitHub). See the Nature Portfolio guidelines for submitting code & software for further information.

Data

Policy information about availability of data

All manuscripts must include a data availability statement. This statement should provide the following information, where applicable:

- Accession codes, unique identifiers, or web links for publicly available datasets
- A description of any restrictions on data availability
- For clinical datasets or third party data, please ensure that the statement adheres to our policy

The source data supporting the findings of this study, including high-dimensional TIFF images, have been deposited at: https://doi.org/10.5281/zenodo.7383719
Publicly available resources used in this study were accessed via: GEO (GSE154795, GSE162631), OSF (doi.org/10.17605/OSF.IO/4Q32E), Human Protein Atlas

(https://www.proteinatlas.org/ENSG00000005381-MPO/immune+cell), The Cancer Genome Atlas (glioblastoma, Firehose Legacy) accessed via cBioPortal (https://www.cbioportal.org), and https://joycelab.shinyapps.io/braintime/ (data from Klemm et al 2020)

Field-specific reporting

Please select the one below that is the best fit for your research. If you are not sure, read the appropriate sections before making your selection.

☒ Life sciences ☐ Behavioural & social sciences ☐ Ecological, evolutionary & environmental sciences

For a reference copy of the document with all sections, see nature.com/documents/nr-reporting-summary-flat.pdf

Life sciences study design

All studies must disclose on these points even when the disclosure is negative.

Sample size	270 samples from 139 primary brain tumor patients and 119 brain metastasis samples from 46 patients were included in our IMC analysis. A subset of the glioblastoma patients used for IMC (n=70; based on tissue availability) plus an additional 65 new glioblastoma patients were used for IHF validation (n=135 in total for IHF). We included all samples we had access to for analyses.
Data exclusions	Exclusion criteria were pre-established for the balanced STS and LTS cohort (depicted in Extended Data Fig. 9f): We excluded patients with unknown IDH status; we excluded tumors representing progression from grade II/III, recurrent or residual disease; we excluded patients that did not receive standard of care, we excluded patients that received a partial (or unknown) resection; and we excluded patients surviving between 1-3 years. Patients with brain metastases from primary sources other than melanoma, breast or lung were excluded from cellular interaction and neighbourhood analyses.
Replication	All antibody optimization was repeated at least 2 times by IHF and 2 times by IMC. All representative immunostaining was performed on ≥5 full tissue sections. All cell types and cellular interactions were identified in multiple patient samples, including replicate samples from the same patient.
Randomization	The glioma patient cohort selected for this study was enriched in long term survivors (>3 years); the brain metastasis patients were not pre-selected for inclusion in this study based on any clinical or histopathological features. Clinical covariates were controlled for the balanced glioblastoma STS and LTS cohort (Extended Data Fig. 9f and Supplementary Table 2).
Blinding	All samples that underwent IMC were stained simultaneously; the order of image acquisition was blinded to clinical data.<br>For all image analyses, quantitative methods were used to eliminate subjective interpretation of data.

Reporting for specific materials, systems and methods

We require information from authors about some types of materials, experimental systems and methods used in many studies. Here, indicate whether each material, system or method listed is relevant to your study. If you are not sure if a list item applies to your research, read the appropriate section before selecting a response.

Materials & experimental systems

n/a	Involved in the study	
☐	☒	Antibodies
☒	☐	Eukaryotic cell lines
☒	☐	Palaeontology and archaeology
☒	☐	Animals and other organisms
☐	☒	Human research participants
☒	☐	Clinical data
☒	☐	Dual use research of concern

Methods

n/a	Involved in the study	
☒	☐	ChIP-seq
☒	☐	Flow cytometry
☒	☐	MRI-based neuroimaging

Antibodies

Antibodies used

Information for all antibodies including clones can be found in Supplementary Table 1. Information for antibodies used for immunofluorescent staining can be found in the Methods. A complete list is below:

Antibody / Clone / Dilution / Catalogue Number / Supplier

IMC Antibodies:
CD3 / Polyclonal / 1:50 / 3170019D / Fluidigm
CD8a / C8-144 / 1:50 / 3162034D / Fluidigm
CD4 / EPR6855 / 1:100 / ab133616 / Abcam
FoxP3 / 236A-E7 / 1:50 / ab20034 / Abcam
CD20 / H1 / 1:100 / 3161029D / Fluidigm
CD94 / EPR21003 / 1:100 / ab235441 / Abcam

CD68 / KP1 / 1:50 / 3159035D / Fluidigm
CD163 / EDHu-1 / 1:200 / 3147021D / Fluidigm
P2Y12/ Polyclonal / 1:100 / AS-55043A / Labscoop
CD11c / EP1347Y / 1:100 / ab52632 / Abcam
HLA-DR / EPR3692 / 1:100 / ab92511 / Abcam
CD14 / SP192 / 1:100 / ab183322 / Abcam
CD16 / SP175/ 1:100 / ab183354 / Abcam
CD117 / YR145 / 1:100 / ab32363 / Abcam
MPO / EPR20257 / 1:100 / ab208670 / Abcam
PanCK / AE1+AE3 / 1:100 / ab80826 / Abcam
PMEL / HMB-45 / 1:100 / NBP2-34638 / Novus Biologicals
MelanA / A103 / 1:100 / sc-20032 / Santa Cruz
Sox2 / EPR3131 / 1:200 / ab215970 / Abcam
Sox9 / EPR14335-78 / 1:100 / ab185966 / Abcam
Olig2 / EPR2673 / 1:200 / ab220796 / Abcam
CD31 / JC-70A / 1:100 / ab9498 / Abcam
GFAP / EP672Y / 1:400 / ab33922 / Abcam
CD45 / D9M8I / 1:100 / 3152018D / Fluidigm
Ki67 / B56 / 1:100 / ab279657 / Abcam
CC3 / 5A1E / 1:100 / 3172027D / Fluidigm
Claudin-5 / EPR7583 / 1:100 / ab131259 / Abcam
Ox40L / EP1168Y / 1:100 / ab76130 / Abcam
MMP9 / EP1255Y / 1:100 / ab137867 / Abcam
M-CSF-R / SP211 / 1:100 / ab183316 / Abcam
GM-CSF-R / 4H1 / 1:50 / 305902 / Biolegend
CTLA-4 / SP355 / 1:100 / ab227709 / Abcam
HIF1α / EP1215Y / 1:100 / ab51608 / Abcam
CD39 / EPR20627 / 1:100 / ab223842 / Abcam
CD40 / EPR20540 / 1:50 / ab213205 / Abcam
pERK / D13.14.4E / 1:100 / 3171010A / Fluidigm
pSTAT3 / EP2147Y / 1:100 / ab76315 / Abcam
CIRBP / EPR18783 / 1:100 / ab238946 / Abcam
CD206 / EPR22489-7 / 1:100 / ab254471 / Abcam
PD-L1/ E1L3N / 1:50 / 13684T / Cell Signaling Technologies
Anti-Biotin / 1D4-C5 / 1:50/ 3150008B / Fluidigm
PD1 / D4W2J / 1:50 / 86163 / Cell Signaling Technologies

Immunohistofluorescence Antibodies:
MPO / EPR20257 / 1:500 / ab208670 / Abcam
IBA1 / Polyclonal / 1:400 / 019-19741 / Fujifilm Wako Pure Chemical Corporation
Horse Anti-Rabbit IgG HRP Polymer Kit / 1:1 / MP-7801 / Vector Laboratories

Validation

All antibodies used in this study were individually titrated for each lot, to determine optimal concentrations.

IMC Antibodies: All in-house conjugated antibodies were validated first by immunohistofluorescence (IHF) imaging prior to conjugating to metal isotopes. IHF staining was validated with secondary-only staining and co-staining for expected overlapping markers. All conjugated antibodies (both in-house and commercially available) were validated by imaging mass cytometry (IMC) based on expected expression pattern in several positive/negative control tissues and co-localization/exclusion with other markers. In all cases, staining specificity was assessed in lymphoid control tissue (tonsil), brain tumor (glioblastoma and/or brain metastasis) and a positive or negative control tissue (such as spleen, liver, kidney, lymph node, normal brain, normal lung, lung cancer). HIF1α was additionally validated using samples from patients with von Hippel Lindau syndrome (positive control) and appendicitis (contained positive and negative cells).

Multiplex IHF Antibodies: Antibody specificity was evaluated via expected cell morphology and co-staining with overlapping markers (ex. IBA1 and CD68). Staining was validated with secondary-only staining.

Human research participants

Policy information about studies involving human research participants

Population characteristics

We collected biobanked tumor samples from patients aged 25 to 96 for IMC. All other information on population characteristics and covariates can be found at https://doi.org/10.5281/zenodo.7383719

Recruitment

We obtained biobanked tumor samples for IMC from primary (glioma) and brain metastasis patients that underwent surgical resection between 2003-2019, and were contacted to give consent for use of their samples in this study. The primary brain tumor cohort was enriched in samples obtained from long-term survivors (> 3 years), therefore, our findings may be biased towards this group.

Ethics oversight

The patient material used in this study was obtained and used in accordance with the following institutional review boards:
1. McGill University cohort: McGill University Health Centre and the Montreal Neurological Institute and Hospital institutional review boards (NEU-10-066, 2018-4150)
2. University of Calgary cohort: Health Research Ethics Board of Alberta, Cancer Committee (HREBA.CC-16-0762)

3. Netherlands Cancer Institute (NKI) cohort: Institutional Review Board of the NKI-AVL and NKI-biobank (CFMPB541)

Note that full information on the approval of the study protocol must also be provided in the manuscript.
